# *Kovacikia euganea* sp. nov. (Leptolyngbyaceae, Cyanobacteria), a new chlorophyll *f* producing cyanobacterium from the Euganean Thermal District (Italy)

**DOI:** 10.3389/fmicb.2025.1545008

**Published:** 2025-03-10

**Authors:** Raffaella Margherita Zampieri, Edoardo Bizzotto, Stefano Campanaro, Fabrizio Caldara, Micol Bellucci, Nicoletta La Rocca

**Affiliations:** ^1^Institute of Research on Terrestrial Ecosystems, National Research Council, Florence, Italy; ^2^Department of Biology, University of Padua, Padua, Italy; ^3^Pietro d’Abano Thermal Studies Center, Padua, Italy; ^4^Science and Innovation Directorate, Italian Space Agency (ASI), Rome, Italy; ^5^National Biodiversity Future Center, Palermo, Italy

**Keywords:** chlorophyll *f*, cyanobacteria, FaRLiP, far-red light, genomics, hot springs

## Abstract

Hot springs are considered modern terrestrial environments analogous to Archean continental surfaces, where photosynthetic life could have evolved. In this habitat cyanobacteria dominate thanks to the adaptations to high temperature and the capability to acclimate to low light intensity and far-red enriched spectra typical of microbial biofilms. The isolation and characterization of new cyanobacterial species from these environments is fundamental to discover genetic and physiological traits allowing them to thrive under such unfavorable conditions, giving useful information to understand the evolution and plasticity of oxygenic photosynthesis as well as to assess their metabolic biodiversity for biotechnological purposes. In this study, we present the polyphasic characterization of a filamentous cyanobacterium, denominated strain ETS-13, isolated from mud biofilms collected in the Euganean Thermal District (Italy). The area is known since ancient times for the presence of thermal springs and muds exploited for the beneficial properties linked to heat, electrolytes, and organic compounds produced by the microbiota. The ETS-13 genome was assembled and annotated, while phylogenetic analyzes were performed using a combined approach based on the 16S rRNA sequence and considering the 16S-23S ITS secondary structures. In addition, morphological, biochemical, and physiological features of the organism were investigated, allowing its classification as a new species of the *Kovacikia* genus, named *Kovacikia euganea*, which formed a cluster with other species of Leptolyngbyaceae from thermal environments. Interestingly, the strain was the first isolated in Italy capable of performing Far-Red Light Photoacclimation (FaRLiP) when exposed to far-red light, a feature found in other species of the same genus so far tested for this acclimation and isolated form geographically distant and different environments.

## 1 Introduction

Thermotolerant and thermophilic cyanobacteria possess remarkable adaptation capabilities that allow them to dominate thick microbial biofilms, visible as dark green, yellow, or orange mats covering surfaces in thermal springs. To thrive in these niches characterized by extreme temperatures and peculiar light conditions, they have evolved high physiological plasticity and adaptability. Photoacclimation is one of the key processes that allows cyanobacteria to proliferate as dominant primary producers within dense microbial biofilms in thermal environments (Mehetre et al., 2022). Microbial mats are layered with billions of cells, and those at the bottom experience different light spectra compared to those at the surface (Gan et al., 2015). Organisms located in lower layers of the mat are visible light-limited due to light scattering, absorption, and refraction, which results in a far-red light enriched niche (Gan et al., 2015). In some cases, cyanobacteria strains have the ability to achieve extensive remodeling of the photosynthetic apparatus and light-harvesting antennae (phycobilisomes), such as Low-Light Photoacclimation (LoLiP) (Zhao et al., 2015), Far-Red Light Photoacclimation (FaRLiP), and Chromatic Acclimation (CA) (Sanfilippo et al., 2019) in species carrying specific genes, which are expressed in response to peculiar irradiation regimes. An important photoacclimation in biofilms is the FaRLiP response. This process was first described in a filamentous cyanobacterium isolated from a floating mat in a hot spring (Yellowstone National Park, USA), *Leptolyngbya* sp. JSC-1 (Brown et al., 2010) and is achieved thanks to a cluster of ~20 genes. A leading role is played by chlorophyll *f*, which supports water oxidation despite using low-energy photons that were thought to be the limit for oxygenic photosynthesis. Only a small number of cyanobacteria species capable of photosynthesize in far-red light have been isolated and characterized so far (Antonaru et al., 2020). These cyanobacteria belong to different genera, are phylogenetically distant and have a punctiform distribution on Earth but have always been found in shaded and far-red light enriched environments such as hot spring mats, microbial mats, desert rocks, soils, and stromatolites (Gan et al., 2015). Both geochemical and biological evidence suggest that cyanobacteria, the first organisms capable of oxygenic photosynthesis, could have evolved on Earth during the Archean in UV-protected ecological niches of hydrothermal and hypolithic habitats where far-red light dominated (Havig and Hamilton, 2019). It is believed that the first forms of oxygenic photosynthesis using far-red light may have played a significant role in Earth’s early history (Gisriel et al., 2022). Hot spring systems such as Yellowstone National Park are, in fact, considered modern terrestrial environments potentially analogous to Archean continental surfaces. The study of their microbial communities represented by biofilms dominated mainly by anoxygenic bacteria and cyanobacteria could help to better understand photosynthetic processes in these peculiar conditions.

Furthermore, to thrive in thermal environments, cyanobacteria synthesize a variety of molecules and secondary metabolites that are needed to support their growth and survival in these harsh conditions (Babele et al., 2019). Therefore, the investigation of these organisms could have biotechnological relevance: they produce bioactive molecules with antibiotic, anti-inflammatory, antioxidant, and antiviral properties, and synthesize pigments and thermostable enzymes (Patel et al., 2018). The cyanobacterium investigated in this study, initially named ETS-13 (where ETS stands for Euganean Thermal Strain), was collected from the Euganean Thermal District, a territory located in the northeast of Italy. The area is known for the presence of thermal springs that have been exploited since ancient times for the beneficial properties of their muds and waters. The territory is characterized by an underground hydrothermal basin from which the water flows to the surface with an average temperature of 75°C, through natural springs, thermal lakes, and artificial wells (Fabbri et al., 2017). Today, nearly 100 spas are present in the area and produce therapeutic muds in special ponds through a codified mud maturation process (see (Gris et al., 2020) for a detailed description of the process). The microbial biodiversity that characterizes this peculiar environment is unique and complex, and only a few of the cyanobacteria found using NGS analysis have been isolated and fully characterized so far (Gris et al., 2020). Some of these organisms were found to be more abundant in mud biofilms at temperatures lower than 45°C (*Phormidium* sp. ETS-05, *Cyanobacterium aponinum* ETS-03) have been already taxonomically characterized (Ceschi Berrini et al., 2004; Moro et al., 2007) and investigated for the production of different compounds of interest that act as anti-inflammatory, antioxidant, and immune modulating agents (Ulivi et al., 2011; Gudmundsdottir et al., 2015; Zampieri et al., 2020, 2022, 2023). Instead, except for *Thermospirulina andreolii* ETS-09 (Moro et al., 2021), little information is available on species that thrive at higher temperatures arranged in dense and thick microbial mats, enriched in filamentous species (Gris et al., 2020). It is indeed from this warmer environment that the species subject of this study was isolated. The filamentous cyanobacterium ETS-13 was collected from two mud ponds at 45°C. A polyphasic approach was used for precise taxonomic classification (Komárek, 2016) since it is considered the most effective method to classify cyanobacteria. Phylogeny, morphology, biochemistry, and physiology were evaluated. Furthermore, ecology was a crucial characteristic evaluated, as reported by Cordeiro et al. (2020), considering the niche in which ETS-13 is growing. To correctly assign the strain to a genus, both the phylogenies of the 16S rRNA gene and the secondary structure analyzes of the 16S-23S ITS region were considered, as they have been shown to be useful for precise taxonomic resolution. Morphology was evaluated along with physiological tests that were performed by exposing the organism to different conditions: temperature, light intensity, and availability of nutrients. The FaRLiP capacity of the strain was also tested. Finally, the genome was assembled, and gene finding and annotation allowed the detection of genomic features of the strain that could be further investigated to understand the metabolic properties of this thermophile.

## 2 Materials and methods

### 2.1 Sampling sites, isolation, and maintenance of the organism

ETS-13 is widespread in the Euganean Thermal District, both in the maturation ponds of the spas and in the natural thermal springs. Detailed information on the physical and chemical parameters of thermal water, muds and microbiota compositions are present in Calderan et al. (2020) and Gris et al. (2020). Two ETS-13 isolates were obtained from biofilms growing at 45 ± 1°C in two different mud maturation ponds of the Euganean Thermal District (Abano Terme, 45° 21′N 11° 46′E). The portions of the microbial mats containing a mixed population of microorganisms, were inoculated in flask in BG11 medium that was exposed to high temperature (60°C) and low light intensity (5 μmol photons m^−2^ s^−1^) for several months; incubation parameters were kept constant until only one strain was present in the flasks. This was identified as ETS-13 by comparing its V4 region sequence of the 16S gene with that obtained by Gris et al. (2020). The isolated organism was then kept in the laboratory under sterile conditions in flasks with BG11 medium (Rippka et al., 1979) that was also used for the experiments.

### 2.2 Experimental setups

Different batch tests were conducted to assess the response of the strains when exposed to varying conditions: temperatures, light intensities and spectra, and nutrient availability. ETS-13 tends to grow in thick biofilms that are difficult to sample, making it impossible to grow under stirring or aerated conditions, causing cell death after a few days. Therefore, ETS-13 was grown in 100 mL flasks with 30 mL of culture or in 6-well plates with a volume of work of 4 mL. Growth was carried out at different temperatures from 30 to 65°C for 7 days with 5 μmol photons m^−2^ s^−1^ of white light (four biological replicates). Inocula for the following different experiments were grown at 30°C with 5 μmol photons m^−2^ s^−1^ of continuous light. Experiments to test responses to different light intensities (30, 60 and 120 μmol photons m^−2^ s^−1^) were carried out for 14 days using white light at 30°C (six biological replicates). Far-red light exposure experiments lasted 21 days under 20 μmol photons m^−2^ s^−1^ for both solar and far-red light at 30°C (four biological replicates). The illumination systems used were white light, True-Light® 18 W T8 solar light simulator (True-Light International GmbH, Kelkheim, Germany) and far-red light LEDs SMD OSLON SSL 80730 nm (Osram Opto Semiconductors GmbH, Regensburg, Germany). Light spectra are indicated in Supplementary Figure S1. Finally, 14-day experiments were also conducted to test the nitrogen fixation capability (three biological replicates). In this case, the inocula were divided after centrifugation and resuspended in fresh medium: BG11 as the control condition and BG11_0_ (Rippka et al., 1979), which lacks NaNO_3_.

### 2.3 Evaluation of growth and *in vivo* absorption spectra

Biomass was evaluated only as fresh and dry weight, due to the formation of thick aggregates. For dry weight measurements, samples collected at the end of the cultivation were placed for 24 h at 50°C. *In vivo* absorption spectra were obtained by centrifugating the organism at 3000 *g* for 5 min. The pellets were gently disrupted with a pestle to disaggregate the cells, resuspended in fresh medium, and analyzed with a spectrophotometer at wavelengths ranging from 350 to 750 nm. Cuvettes were placed with the opaque side oriented toward the ray, as described in Gan et al. (2014).

### 2.4 Extraction and quantification of lipophilic and hydrophilic pigments

Lipophilic extracts were obtained by resuspending the samples in N,N-dimethylformamide. After 24 h at 4°C and dark conditions, disrupted cells were centrifuged, and the supernatant was measured from 350 to 750 nm at the spectrophotometer (Zampieri et al., 2023). For hydrophilic extracts, saline phosphate buffer (0.15 M NaCl, 0.01 M Na_2_HPO_4_) was used together with glass beads (150–212 nm, Sigma-Aldrich, St. Louis, Missouri, USA) to disrupt cells. Cell disruption cycles using a bead-beater for 30 s, alternated to 30 s on ice, were performed three times. The supernatant collected after centrifugation was analyzed with a spectrophotometer at wavelengths ranging from 350 to 750 nm. Pigment content was quantified using the following formulas (Bennett and Bogorad, 1973; Chamovitz et al., 1993):


$$\mathrm{Chlorophyll}\;a\;\left(\mathrm{\mu g}\;{\mathrm{mL}}^{-1}\right)=A_{664}\times 11.92$$

$$\mathrm{Carotenoids}\;\left(\mathrm{\mu g}\;{\mathrm{mL}}^{-1}\right)=A_{461}-\left(0.046\times A_{664}\right)\times 4$$

$$\mathrm{Phycocyanin}\;\left(\mathrm{mg}\;{\mathrm{mL}}^{-1}\right)=\left[A_{615}-\left(0.474\times A_{652}\right)\right]/5.34$$

$$\mathrm{Allophycocyanin}\;\left(\mathrm{mg}\;{\mathrm{mL}}^{-1}\right)=\left[A_{652}-\left(0.208\times A_{615}\right)\right]/5.09$$

The dry weight of the samples was measured before extraction to correlate the concentration of pigments with the biomass. No differences in the quantity and quality of pigments were observed when extracted from dried and undried samples (data not shown). Therefore, the amounts are reported as mg g_DW_^−1^.

### 2.5 HPLC analysis

Pigments extraction was performed using 90% acetone (in water suitable for HPLC) alternating 4 cycles of cell rupture in a bead beater using 150–212 nm diameter glass beads and 30 s in ice. The supernatant was collected after centrifugation and used for analysis. Pigment content was determined using Agilent 1,100 series high-performance liquid chromatography (HPLC) Agilent 1,100 series LC system (Agilent Technologies, Santa Clara, California, USA), equipped with a reversed-phase column as reported in Gris et al. (2017). For the FaRLiP analysis, the protocol reported by Battistuzzi et al. (2023) was used.

### 2.6 Microscopy analysis

Optical microscopy images were acquired using a Leica DM6B microscope (Leica Microsystems, Wetzlar, Germany), equipped with a Leica DFC7000T camera. Cell size measurements were performed using ImageJ software (v. 1.52a) (Schneider et al., 2012). The filament staining was carried out using Alcian Blue in 3% acetic acid, pH 2.5 to visualize polysaccharidic matrix. Samples for transmission electron microscopy (TEM) analysis were prepared as described in Fattore et al. (2021).

### 2.7 Genome extraction and sequencing

Genomic DNA was extracted from ETS-13 pelleted cells resuspended in 200 μL of TEN cold extraction buffer (100 mM Tris–HCl pH 8; 50 mM EDTA pH 8; 500 mM NaCl). Approximately 50 μL of acid-washed sterile glass beads (150–212 μm) was added and the cells were disrupted using a bead-beater with 5 cycles of 30 s. A total 35 μL of 20% SDS were supplemented, and the sample was heated at 65°C for 5 min, after adding 130 μL of 5 M KOAc the tube was placed on ice and centrifuged. The supernatant was gently collected in a new tube and DNA was precipitated after adding 500 μL of ice-cold isopropanol and a 10-min incubation at −20°C. The pellet obtained after centrifugation was carefully washed with EtOH 70% twice. The sample was then left to dry and finally resuspended in DEPC-treated water. Recovery of DNA from pellet and supernatant samples was ensured by qualitative and quantitative analyzes on the samples, using a NanoDrop Microvolume UV–Vis spectrophotometer (Thermo Fisher Scientific, Waltham, Massachusetts, USA) and a Qubit Fluorometer (Thermo Fisher Scientific). DNA samples underwent library preparation using the Nextera DNA Flex Library Prep Kit (Illumina Inc., San Diego, California, USA). The libraries were sequenced using the Illumina Novaseq platform. Nanopore sequencing was performed using the Rapid Barcoding Kit (SQK-RBK004) and a FLO-MIN106 R9 flow cell on a MinION device (Oxford Nanopore Technologies, Oxford, UK). All libraries were prepared at the CRIBI Biotechnology Center sequencing facility (University of Padova, Italy). The assembly was obtained from Nanopore and Illumina reads. Nanopore reads were assembled using Canu (v. 2.2) (Koren et al., 2017). Illumina reads were trimmed with Trimmomatic (v. 0.39) (Bolger et al., 2014) and aligned on assemblies using Bowtie2 (v. 2.4.4) (Langmead and Salzberg, 2012) to polish the Nanopore assembly using Pilon (v. 1.24) (Walker et al., 2014). Genome quality was assessed using CheckM (v. 1.1.3) (Parks et al., 2015). Genes were predicted using Prodigal (v. 2.6.3) (Hyatt et al., 2010) and annotated with eggNOG (v. 2.1.2) (Cantalapiedra et al., 2021). 16S rRNA sequences were predicted using Barrnap (v. 0.9) (Seemann, 2025). Circular genome representation was obtained using the Artemis DNAplotter module (Li et al., 2016) as previously described (Campanaro et al., 2014). Raw reads were deposited in the NCBI Sequence Read Archive Database (SRA) under the BioProject PRJNA946549.

### 2.8 Phylogenetic analysis

Evolutionary analyzes were performed in MEGA11 (v. 11.0.13) (Tamura et al., 2021). Two separate datasets were constructed for the 16S gene and the 16S-23S ITS region using the sequences of ETS-13 and other relevant species identified through BLAST N search or strains of the Leptolyngbyaceae family. Datasets for analysis were aligned with MEGA11 Muscle tool using default options. Multiple sequence alignment was visualized using alignment viewer software^1^ coloring columns with conservation higher than 0.9 (Supplementary Figure S2). The evolutionary history of the 16S rRNA gene, which involved 41 nucleotide sequences and a total of 1,108 positions in the final dataset, was inferred by using the Maximum Likelihood (ML) method and General Time Reversible (GTR) model (Nei and Kumar, 2000). Initial trees for the heuristic search were obtained automatically by applying Neighbor-Join and BioNJ algorithms to a matrix of pairwise distances estimated using the Maximum Composite Likelihood (MCL) approach. This analysis was performed with the following parameters: Gamma distribution (4 categories (+*G*, parameter = 0.1713) [+*I*], 37.14% sites). The evolutionary history was inferred also using the Neighbor-Joining (NJ) method (Saitou and Nei, 1987). The evolutionary distances were computed using the Kimura 2-parameter method (Kimura, 1980). The rate variation among sites was modeled with a gamma distribution (shape parameter = 1). All positions containing gaps and missing data were eliminated (complete deletion option). The Maximum Parsimony method was conducted under these parameters: the consistency index of 0.382326 (0.335335), the retention index of 0.656670 (0.656670), and the composite index of 0.251062 (0.220205) for all sites and parsimony-informative sites (in parentheses). The MP tree was obtained using the Subtree-Pruning-Regrafting (SPR) algorithm (Nei and Kumar, 2000).

The evolutionary history of the 16S-23S ITS region, which involved 23 nucleotide sequences and 786 positions, was inferred by using the ML method and the GTR model (Nei and Kumar, 2000). A discrete Gamma distribution was used to model evolutionary rate differences among sites [10 categories (+*G*, parameter = 0.4668)]. The rate variation model allowed some sites to be evolutionarily invariable ([+*I*], 17.30% sites). The evolutionary history was inferred, as described before, using the NJ and MP methods [consistency index: 0.573294 (0.512873), retention index: 0.509215 (0.509215), composite index: 0.291930 (0.261162)].

The secondary structure of the ITS region was extracted based on findings from a previous work (Iteman et al., 2000) for the D1-D1’ and boxB helices. Secondary structures of RNA molecules were predicted using mfold RNA Folding Form V2.3 with default parameters and “Untangle with loop fix” as structure draw mode (Zuker, 2003).

### 2.9 Identification of relevant genes and statistical analysis

BLAST N and tBLAST N were used to identify orthologous genes in ETS-13 genome. FaRLiP gene clusters were created using BioRender.com. Statistical analyzes were performed using GraphPad Prism (9.4.1, GraphPad Software, USA). The tests used are indicated for each experiment in the results: Ordinary One-way ANOVA with Tukey’s multiple comparison test, two-way ANOVA with Šídák’s multiple comparisons test, followed by a two-tailed unpaired *t*-test was applied when only two groups were compared. *p*-values are reported in figures.

## 3 Results and discussion

### 3.1 16S rRNA gene phylogeny

**Figure 1:**
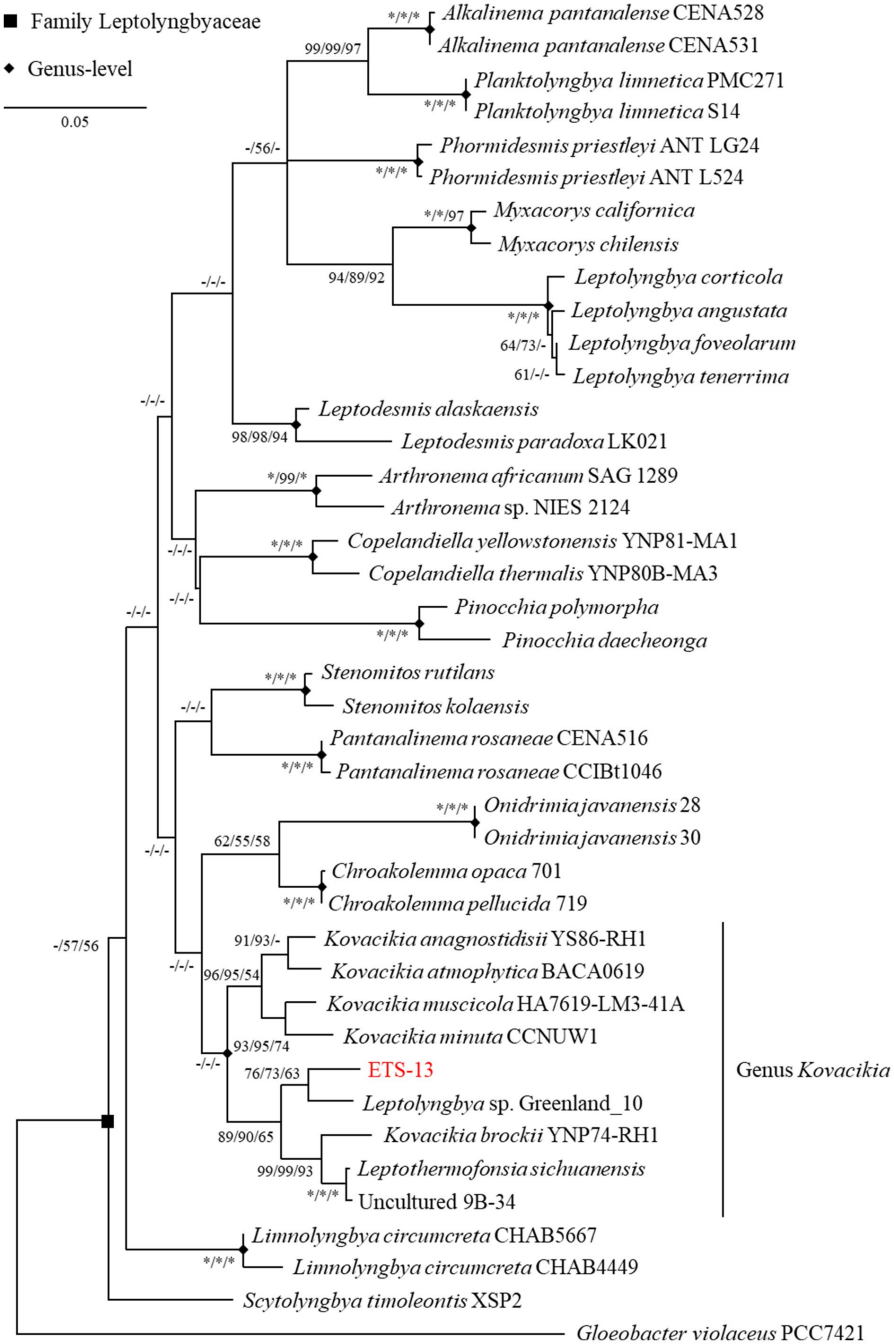
ML phylogenetic tree of 16S gene sequence inferred using GTR model. The tree with the highest log likelihood (−6629.47) is shown. The tree is drawn to scale, with branch lengths measured in the number of substitutions per site. The percentage of replicate trees in which the associated taxa clustered together in the bootstrap tests (1,000 replicates each) are reported close to the nodes as ML/NJ/MP (* equals to 100, − if percentage is lower than 50).

Phylogenetic analysis was performed considering the 16S rRNA gene sequence (Supplementary Table S1 and Supplementary Figure S2). The highest similarity (96.24%) was found with *Leptolyngbya* sp. Greenland 10, a strain isolated from hot springs in Greenland, but lacking detailed characterization (Roeselers et al., 2007). This strain was formerly attributed to the genus *Leptothermofonsia* (Tang et al., 2022), however this genus was later merged to *Kovacikia* as reported by Kaštovský et al. (2023). The second hit of the BLAST N analysis (95.42%) was with the *Leptothermofonsia sichuanensis* strain PKUAC-SCTE412 (Tang et al., 2022) isolated from a hot spring in Sichuan province, China. Following, the sequence matched with *Kovacikia anagnostidisii* (95.35% of similarity), from Clearwater springs in Yellowstone National Park, USA The fourth result gave a 95.24% identity with an uncultured cyanobacterium (clone 9B-34) for which the only available information is the isolation site, a hot spring mat in Yunnan province, China. The high similarity of this last strain with *Leptothermofonsia sichuanensis* (at least in terms of 16S rRNA gene sequences) suggested that they are indeed isolates of the same species. Identity values ranging from 94.5 to 93.5% were obtained with other genera of Leptolyngbyaceae, including other species of the genus *Kovacikia, Stenomitos*, *Chroakolemma*, *Leptodesmis,* and *Pantanalinema*. These indications suggested a possible assignment of ETS-13 isolate to the *Kovacikia* genus, considering also the typically used threshold of 95% of identity to assign a species to a genus (Schloss and Handelsman, 2005). According to the assumption that sequences having more than 97% identity are frequently representative of the same species (Johnson et al., 2019), ETS-13 was recognized as a new species of the *Kovacikia* genus. Data were confirmed by ML phylogenetic analysis (Figure 1) that was performed considering other genera of the Leptolyngbyaceae family and using *Gloeobacter violaceus* as an outgroup. The same dataset was analyzed using the NJ and the MP tests, resulting in a topology similar to the ML tree. ETS-13 was included in the “*Kovacikia* genus clade” together with the other recognized species *K. brockii*, *K. minuta*, *K. muscicola*, *K. atmophytica*, *K. anagnostidisii* as well as *Leptothermofonsia sichuanensis* and *Leptolyngbya* sp. Greenland 10. The uncultured cyanobacterium clone 9B-34 was also integrated into the genus with solid bootstrap values. This result, together with the identity values obtained from the similarity search, supports the suggested hypothesis that the genus has a cosmopolitan distribution and that many of its members are specific to thermal environments (Tang et al., 2022).

### 3.2 16S-23S ITS phylogeny and secondary structures

Further phylogenetic investigations were conducted considering the 16S-23S ITS region. Analysis was done, as for the 16S rRNA gene, including genera of the Leptolyngbyaceae family. The number of sequences reported in public databases was smaller than 16S rRNA genes, clearly evidenced by the number of species in the ML tree (Supplementary Figure S3). It is known that the use of the 16S-23S ITS region for phylogenetic analysis can provide reliable results only when very closely related strains are considered (Johansen et al., 2011), while in other cases it is likely to represent an inconsistent method due to the great interspecies heterogeneity of the ITS region. For this reason, phylogenetic analysis based on the 16S-23S ITS region may lead to inaccurate taxonomic classification. However, the obtained topology was verified by NJ and MP tests, with ETS-13 clustering in the *Kovacikia* genus. Other genera considered clustered as expected with high bootstrap values that confirmed the results obtained from 16S rRNA. Moreover, the analysis of the secondary structures of this region provided another important contribution to the final determination of the taxonomy. Regarding the D1-D1’ region (Supplementary Figure S4a), ETS-13 presents the same pattern in terms of interior loops and region length with phylogenetically close species, representing a strong indication among the members of the *Kovacikia* genus. In the boxB region (Supplementary Figure S4b), ETS-13 has fewer nucleotides than the other species (39 bp), but similar internal structures can be found in *Kovacikia brockii*. The other three species considered for comparison resulted in a more comparable region in terms of loops and number of nucleotides.

### 3.3 Morphological and biochemical features of strain ETS-13

**Figure 2:**
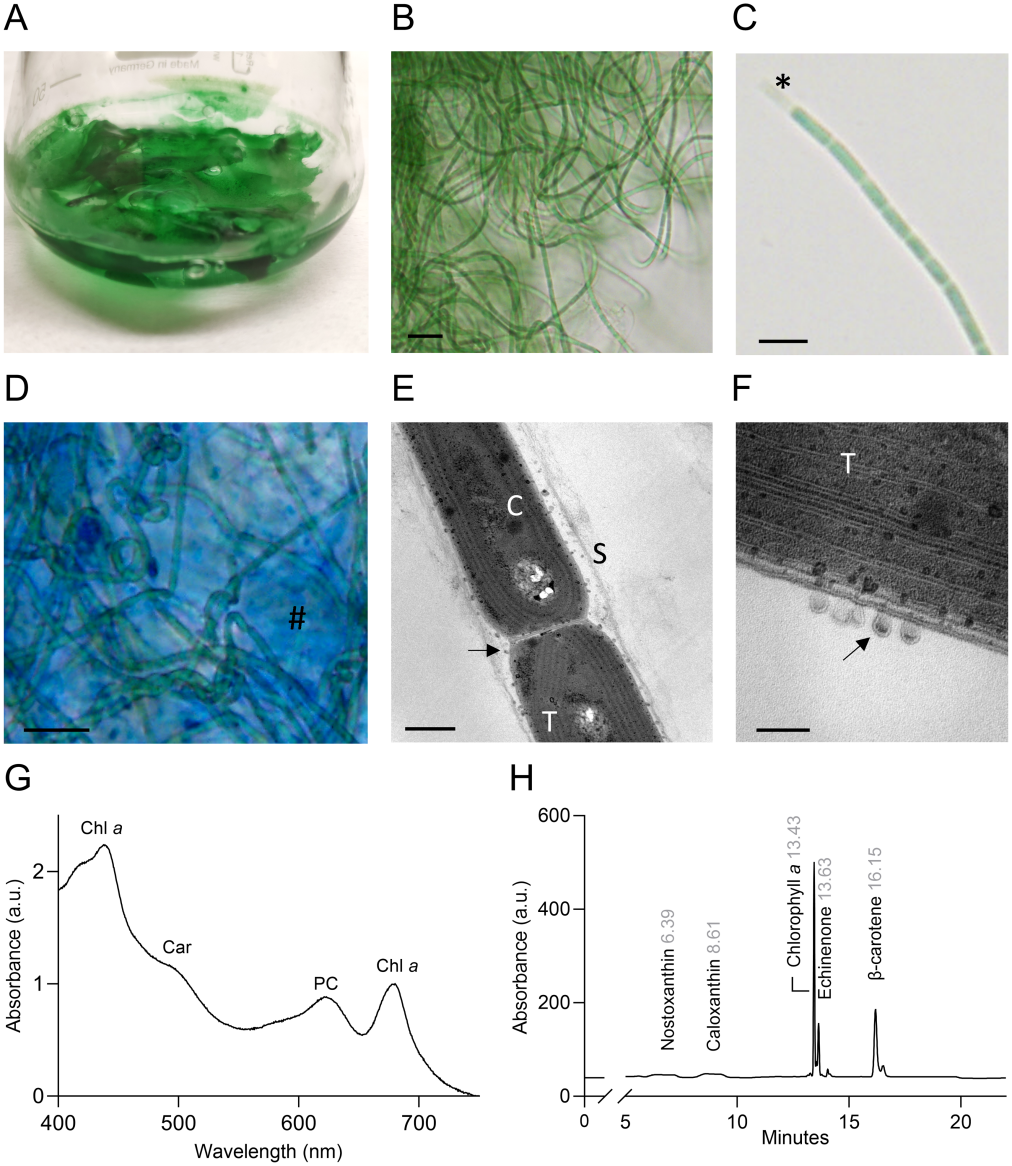
Morphological and biochemical features of ETS-13 **(A)** Culture forming dense and packed floating biofilms. **(B)** ETS-13 filaments largely tangled at light microscopy. Bar 10 μm. **(C)** Trichome with elongated cells. Sheath is highlighted (*) in one extremity. Bar 5 μm. **(D)** Alcian blue coloration highlights cells surrounded by a polysaccharidic matrix (#) Bar 10 μm. Images acquired at TEM **(E)** highlight cell morphology (bar 500 nm) **(F)** and in detail cell membranes and extracellular vesicles (bar 100 nm). S, sheath; T, thylakoids; C, carboxysome, black arrows, extracellular vesicles. **(G)** *In vivo* absorption spectrum normalized at 680 nm. Chl *a*: chlorophyll *a*; Car: carotenoids; PC: phycocyanin. **(H)** HPLC chromatogram of the lipid-soluble pigment extract, retention time is reported in gray.

Morphological analysis revealed filaments that formed mats floating on the surface or deposited at the bottom of the flasks, according to the growth condition (Figure 2A). Light microscopy analysis showed thin green trichomes tangled and coiled in clusters, rarely straight and solitary (Figure 2B). The filaments were isopolar, unbranched, not attenuated at their ends. They were surrounded by a thin colorless sheath that can be observed at the extremity of the fragmented filament in Figure 2C (Kumar et al., 2018). Biofilms of ETS-13 were characterized by highly abundant extracellular polysaccharides, which create a mucilaginous matrix in which filaments are embedded. These were highlighted by the coloration of the culture with alcian blue (Figure 2D), a cationic dye that specifically binds negatively charged polysaccharides (appearing in blue) (Borjas Esqueda et al., 2022). The presence of heterocysts, akinetes, and hormogonia was not observed. Cells observed at TEM appear to be elongated, 1.5 ± 0.1 μm in width, and 2.4 ± 1 μm in length (Figures 2E,F). Thylakoids were set in a simple parietal arrangement, with 4–6 thylakoids parallel to each other in the periphery of the cells. This disposition represents a distinctive characteristic of species belonging to the Synechococcales order (Mareš et al., 2019). Numerous extracellular vesicles were detected between outer membranes and sheaths, with dark or light inclusions suggesting a variable composition of internal molecules. Morphological traits among phylogenetically close genera were compared: *Kovacikia*, *Stenomitos,* and *Chroakolemma* (Table 1). Common characteristics of the *Kovacikia* genus (Miscoe et al., 2016) were verified, including, for example, unbranched filaments, coiled or entangled, cylindrical cells 1–1.5 μm in width, providing additional confirmation on the attribution of ETS-13 to this genus. A distinctive feature of ETS-13 is the stable bright green color, more similar to the pale blue-green pigmentation of *Chroakolemma opaca* and clearly distinct from the brownish color of the other species considered. A darker coloration was observed only in response to high light intensities. Despite the phenotypic characteristics mentioned above, species-level assignment cannot be based solely on morphology, due to the great phenotypical plasticity of this group of Synechococcales (Mai et al., 2018). Analysis of the pigment composition was performed for both the lipophilic and hydrophilic components. The *in vivo* absorption spectrum (Figure 2G) highlighted the presence of phycocyanin and the absence of phycoerythrin, confirmed by the evaluation of the extract of phycobiliproteins composed of 75–80% phycocyanin and 20–25% allophycocyanin. This trait differentiated ETS-13 from *Leptothermofonsia sichuanensis* (Tang et al., 2022) and *Kovacikia minuta* (Shen et al., 2022) that possess phycoerythrin as well. As regards carotenoids, *β*-carotene was the main carotenoid, followed by echinenone (Figure 2H). Smaller amounts of nostoxanthin and caloxanthin were also detected.

**Table 1:** Outline of morphological and habitat features of ETS-13 compared to other species of the family Leptolyngbyaceae.

Species	References	Morphology	Cell width (μm)	Cell length (μm)	Sheaths	Color	Location/Habitat
ETS-13	This study	Tangled, coiled	1.4–1.6	1.4–3.4	Thin, colorless	Bright green	Mud maturation pond (Italy) Thermal
*Leptothermofonsia sichuanensis*	Tang et al. (2022)	Tangled, coiled, closely packed	0.8–1.1	1.2–1.8	Colorless	Brown or green	Lotus Lake hot springs (China) Thermal
*Kovacikia anagnostidisii*	Kaštovský et al. (2023)	Tangled, slightly coiled	1.6–2.0	1–2.1	Thin, colorless	Green or yellow-green	Yellowstone National Park (USA). Clearwater springs. Thermal
*Kovacikia atmophytica*	Luz et al. (2023)	Tagled, straight, slightly waived	1.0–1.6	1.1–2.9	Thin, colorless	Blue-green or greenish to brownish	Terceira Island (Portugal). Furnas do Enxofre. Fumarole
*Kovacikia brockii*	Kaštovský et al. (2023)	Straight, coiled, sometimes spiral	1.1–2.5	0.8–2.2	Thin, colorless	Gray-green or blue-green	Yellowstone National Park (USA). Firehole Lake.Thermal
*Kovacikia minuta*	Shen et al. (2022)	Coiled into clusters	1.1–1.3	0.9–2.2	Thin, colorless, often absent	Purplish brown or green	Wuhan Botanical Garden (China). Pond
*Kovacikia muscicola*	Miscoe et al. (2016)	Straight	1.5–1.7	1.0–2.0	Thin, colorless, often absent	Grayish green to purplish brown	Kauai, Hawaii (USA). Cave walls
*Stenomitos rutilans*	Miscoe et al. (2016)	Bent, entangled	0.9–1.1	2.5–5.0	Absent	Red brownish	Kauai, Hawaii (USA). Cave
*Chroakolemma opaca*	Becerra-Absalón et al. (2018)	Intricate, sometimes coiled	1.0–3.8	1.6–6.9	Thin, colorless, or thick, opaque	Pale blue-gren	Hidalgo (Mexico). Semi-desert soil

### 3.4 Assessment of ETS-13 growth at different temperatures

**Figure 3:**
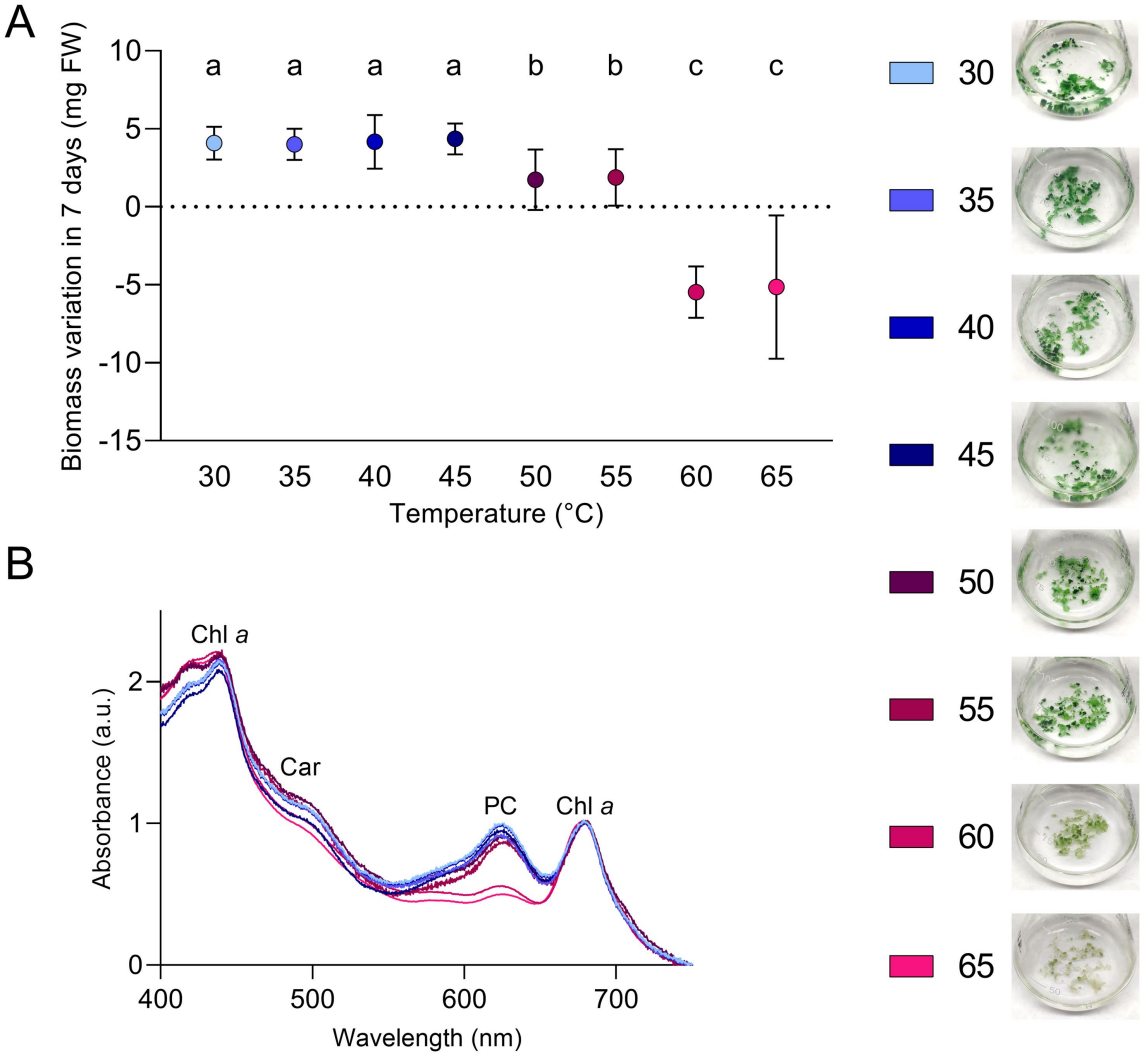
Growth of ETS-13 at different temperatures (*n* = 4). **(A)** Variation in biomass after 7 days of cultivation for each temperature tested measured as fresh wight (FW) with the cultures at the end of the experiments. Statistically significant data are indicated by different letters as follow, a: *p* < 0.001; b: *p* < 0.01; c: *p* < 0.05. **(B)** *In vivo* absorption spectra after 7 days of exposure to the increasing temperatures; data are normalized according to chlorophyll *a* peak at 680 nm. Only one replica for condition is reported. Chl *a*, chlorophyll *a*; Car, carotenoids; PC, phycocyanin.

A range of light and temperature conditions was tested to understand the optimal growth parameters and the limiting ones for this strain. Low light intensity (5 μmol photons m^−2^ s^−1^) was used to detect differences in growth determined only by the temperature parameter. A comparable growth was measured in the range between 30 and 45°C; at these temperatures, the biomass obtained was the highest among the conditions tested (Figure 3A). Considering this range of temperatures as optimal, ETS-13 could be ascribed to high thermotolerant bacteria. Although ETS-13 can grow at 50 and 55°C, the biomass generated in 7 days by the organism was lower. At 60 and 65°C the organism showed a reduction in biomass, as can be clearly seen from the aspect of the cultures that created thinner aggregates with diminished pigmentation, suggesting that high temperatures are not favorable for cell growth. *In vivo* absorption spectra led to similar results (Figure 3B), with no significant differences from 30 to 55°C in relative peaks of phycocyanin, carotenoids, and chlorophyll *a*. At 60 and 65°C a decrease in phycobiliprotein levels was measured, as also confirmed by the lighter color of the cultures. Interestingly, through NGS analysis, the strain was already found as one of the main players in the mud maturation process (Gris et al., 2020) (indicated in the article as OTU-15 Leptolyngbyaceae sp. ETS-13), mainly when temperatures were higher than 47°C and was the most abundant cyanobacterium detected at 54.7°C. It is possible to speculate that in this environment, when the temperature range is between 47 and 55°C, the species is dominant due to a minor competition with the other cyanobacterial species that are characterized by higher growth rates at lower temperatures. The two closely related strains *Leptolyngbya* sp. Greenland 10 and *Leptothermofonsia sichuanensis* were found at slightly higher temperatures: respectively 56–61°C (Roeselers et al., 2007) and 67.2°C (Tang et al., 2022), suggesting that a different level of thermotolerance is found among members of the genus.

### 3.5 Response of ETS-13 to different light intensities

**Figure 4:**
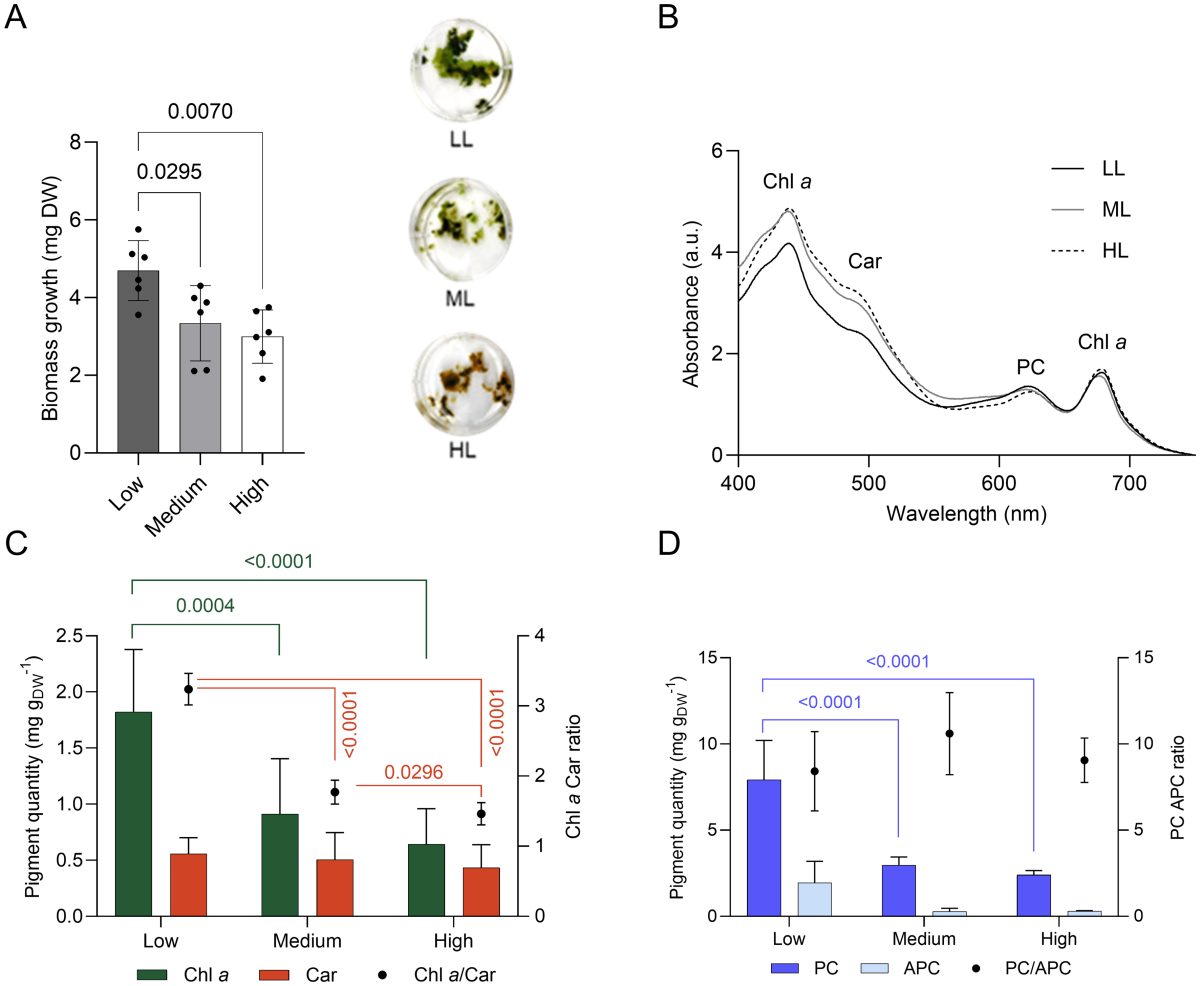
Growth of ETS-13 under increasing light intensities: low (LL), medium (ML), and high (HL) light (*n* = 6). **(A)** Biomass measured as dry weight (DW) with the cultures at the end of the experiment. One-way ANOVA with Tukey’s multiple comparison tests, *p*-values are reported for each experiment if <0.05. **(B)** *In vivo* absorption spectra normalized at 680 nm, **(C)** lipid-soluble and **(D)** water-soluble pigments quantification. Chl *a*, chlorophyll *a*; Car, carotenoids; PC, phycocyanin; APC, allophycocyanin.

The growth of ETS13 cultures was tested for 14 days at increasing light intensities defined as low, medium, and high (respectively 30, 60, and 120 μmol photons m^−2^ s^−1^). The maximum amount of biomass was obtained at low light (Figure 4A) and progressively lower amounts at medium and high light. Cell pigmentation is clearly influenced by light intensity, turning from green in low light to brown in high light. This difference could be highlighted in the *in vivo* absorption spectra (Figure 4B), with an increase in the carotenoid region and a decrease in the peak of phycocyanin in high light compared to low light. Quantification of lipophilic (Figure 4C) and hydrophilic pigments (Figure 4D) confirmed the reduction of both categories at increasing light intensities. The carotenoid content remained stable, leading to a 50% decrease in the ratio of chlorophyll *a* and carotenoids at medium and high lights. Phycocyanin was also halved under medium and high light conditions compared to low light condition. The optimal growth condition found, corresponding to the lower light intensity tested, fits well with the properties of the environment from which the organism was isolated. In fact, ETS-13 grows in thick microbial mats where shading, caused by both other microorganisms and by the turbidity of the thermal water layer, is an important factor to consider and can explain the adaptation of the strain to low light.

### 3.6 Exposure of ETS-13 to far-red light and photoacclimation response

**Figure 5:**
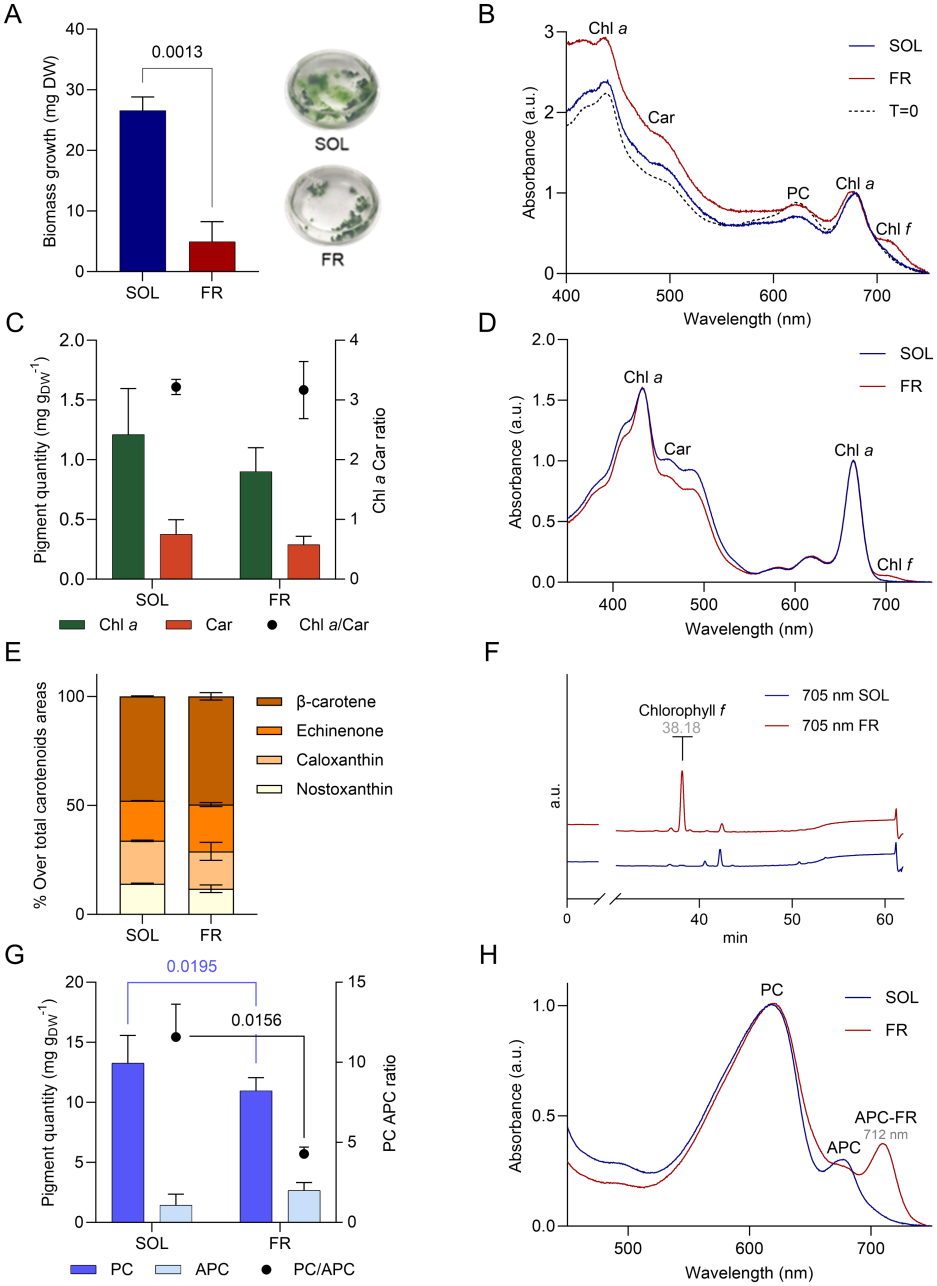
Growth of ETS-13 under solar light (SOL, in blue) and far-red light (FR, in red) for 21 days (*n* = 4). **(A)** Biomass growth measured as dry weight (DW) with the cultures at the end of the experiments and **(B)** *in vivo* absorption spectra normalized at 680 nm comparing starting point (T = 0) and samples collected after 21 days. **(C)** Chlorophyll *a* (Chl *a*) and carotenoids (Car) quantified (left axis) and their ratio (right axis). **(D)** Lipid-soluble extract spectra normalized at 664 nm. **(E)** Relative percentage of each carotenoid detected through HPLC analysis for both conditions. **(F)** HPLC chromatograms at 705 nm of ETS-13 grown for 21 days under solar light (SOL) or far-red light (FR). Retention time of chlorophyll *f* is reported in gray **(G)** Quantification of phycocyanin and allophycocyanin content referred to dry weight is reported on the left axis, while their ratio is on the right axis. **(H)** Phycobiliprotein extract spectra normalized at 615 nm. Statistical analysis: two-way ANOVA with Šídák’s multiple comparisons test and unpaired t test with Welch’s correction (two conditions compared). All spectra are represented only by a single replicate for exemplification. Chl *a*, chlorophyll *a*; Car, carotenoids; PC, phycocyanin; APC, allophycocyanin; APC-FR, far-red shifted allophycocyanin.

The organism was exposed to far-red or solar light for 21 days, starting from an inocula adapted to solar light. Biomass growth, measured as dry weight (Figure 5A), was impaired when ETS-13 was exposed to far-red light, as can be clearly seen looking at the macroscopic aspect of the cultures at the end of the experiment. However, ETS-13 was able to grow using only far-red light, even if at a slower rate, reaching at the end a biomass 8 times lower compared to cells cultivated in solar light. No differences were also detected at the microscopic level considering the length of the filaments or the cell size (Supplementary Figure S5). The *in vivo* absorption spectrum (Figure 5B) maintained a similar shape from the starting point to the end of the experiment in solar light, while the presence of a peak at wavelengths higher than 700 nm was detected in far-red light. This result indicated the activation of the FaRLiP response in ETS-13 grown under far-red light. No significant differences were observed between the two lights for chlorophyll *a* and carotenoids content, leading also to a comparable ratio between the two (Figure 5C). Interestingly, the spectra of the cell extract obtained in far-red light (Figure 5D), did not reveal a peak determined by chlorophyll *f* (700–710 nm) as high as expected from the *in vivo* spectra. To confirm the synthesis of chlorophyll *f* and to assess the entire photosynthetic pigment composition of solar and far-red light acclimated cultures, HPLC analysis was conducted. Different carotenoids showed comparable relative abundances in the two samples, without the synthesis of new types of carotenoids due to far-red exposure (Figure 5E). The presence of the pigment involved in the FaRLiP response was highlighted only in cultures exposed to far-red light as expected. The presence of chlorophyll *f* was evidenced (Figure 5F). However, a large contribution to the 700–710 nm peak detected in the *in vivo* spectrum of ETS-13 exposed to far-red light was determined by phycobiliproteins and in particular by the far-red shifted form of allophycocyanin. This was highlighted in the spectrum of the phycobiliprotein extract of far-red light cultivated cells, indicating a leading role of this pigment in FaRLiP for ETS-13 (Figure 5H). The contribution of far-red shifted allophycocyanin was not quantified since the applied formula does not allow to calculate this form, leading to the possibility that total allophycocyanin in far-red light could have reached even higher values. The overall phycocyanin content was higher in solar light compared to far-red light, while no statistical differences were measured for allophycocyanin (Figure 5G). Finally, the PC/APC ratio was halved in far-red light with respect to solar light, as a result of the diminished PC content in far-red light. The ability of ETS-13 to perform the FaRLiP response supports the hypothesis that this adaptation might be a distinctive feature of the genus *Kovacikia*, with *Kovacikia euganea* as the first species isolated in Italy. In fact, all other *Kovacikia* species tested for FaRLiP, *K. minuta* isolated from shadowed freshwater ponds in Wuhan (China) (Shen et al., 2022) and *L. sichuanensis* isolated from thermal springs in Ganzi (China) resulted also able to grow using far-red light (Tang et al., 2022). Further studies on other organisms within the genus are needed to establish FaRLiP as a common trait of *Kovacikia* species. Since the description of the FaRLiP response in 2014, an increasing number of cyanobacteria have been discovered exhibiting this adaptation, but it has never been identified in all species within a genus. A list of FaRLiP strains is reported in Supplementary Table S2, for about half of them the far-red photoacclimation capability was predicted only using bioinformatics and based on the presence of the FaRLiP gene cluster (Gan et al., 2015) or using the marker gene apcE2 (Antonaru et al., 2020).

### 3.7 Genomic features and identification of relevant genes in ETS-13 genome

**Figure 6:**
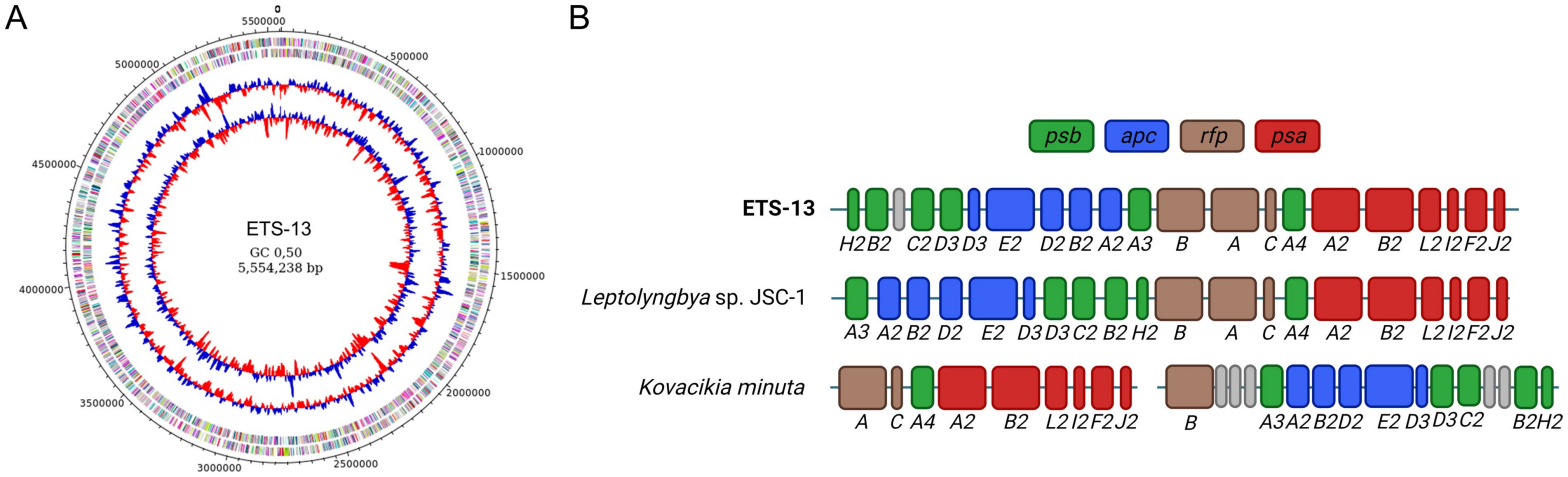
Genomic characteristic of the ETS-13 isolate. **(A)** Genomic representation showing from outside inward: predicted coding sequences (CDSs) localized on forward/reverse strands and colored according to the clusters of orthologous groups (COG), GC skew, and GC content (%). **(B)** Structure of the FarLiP gene cluster of ETS-13, compared to *Leptolyngbya* sp. JSC-1 and *Kovacikia minuta*. In dark green are highlighted the *psb* genes for core subunits of photosystem II, in blue *apc* genes for phycobiliproteins core subunits, in brown phytochrome photoreceptor and regulators genes (*rfp*), and in red *psa* genes for photosystem I core subunits. A hypothetical protein of unknown function is reported in gray. The gap in *Kovacikia minuta* indicates truncated operons in the cluster.

The assembly of ETS-13 consisted of a single closed circular chromosome having a total length of 5,554,238 bp and 49% GC content (Figure 6A). Genome coverage, based on both Illumina and Nanopore reads, is 185 X, a level high enough to ensure a solid quality of the chromosome structure and the predicted sequence. The completeness was assessed by checking for the presence of marker genes with checkM and calculated as 100% with a negligible contamination level of 0.28%. A total of 4,535 genes were identified and annotated with their corresponding COG codes (Supplementary Table S3). The presence of genes and their annotation can provide numerous evidence on the biochemical characteristics of an isolate. Not only gene presence, but also their organization in clusters can be informative, since encoded proteins could work synergistically within a cluster. Cyanobacteria are known to produce a wide variety of metabolites, among which toxic compounds, called cyanotoxins, have previously been reported for many species, which can be species-specific (Dulić et al., 2022). In the present study, the cyanotoxins investigated were microcystin, saxitoxin, cylindrospermopsin, and lyngbyatoxin-A (Supplementary Table S4). No similarity was found with the *mycE* gene (encoding microcystin synthase E) that is involved in microcystin synthesis, nor with *cyrA*, *cyrB*, *cyrC*, *cyrJ* involved in the synthesis of cylindrospermopsin and *sxtA*, *sxtB*, *sxtI* for the production of saxitoxin (Ribeiro et al., 2020). Low levels of sequence identity (20–40%) were obtained for *ltxA*, *ltxB*, and *ltxD*, which are linked to the synthesis of lyngbyatoxin-A (Edwards and Gerwick, 2004), *ltx* gens are not organized as a gene cluster in the ETS-13 genome, and no similarity was found for *ltxC*. Taken together, these results suggest that the strain should not be able to produce cyanotoxins. As for the capability to fix atmospheric nitrogen, the gene cluster responsible was not identified in ETS-13 (Supplementary Table S5) as expected from the growth test performed. The genes necessary for nitrogenase synthesis (Yoon et al., 2017), *nifD* and *nifK*, did not show any similarity within the genome. For other genes related to nitrogenase biosynthesis and related functions, low similarity (ranging from 21 to 56%) of the sequences was observed, but the genes are not organized in a cluster. The ability to fix atmospheric nitrogen was also experimentally tested as reported in Supplementary Figure S6, confirming that ETS-13 is not capable of performing this process. Different photoacclimation processes were investigated: the FaRLiP response, first characterized in *Leptolyngbya* sp. JSC-1 (Gan et al., 2014), is determined by a cluster of 20 genes (Supplementary Table S6), which is responsible for the paralogous core subunits of the photosynthetic complexes (*psb*, *psa*, and *apc* genes) and three regulatory components (*rfp* genes). The presence of the cluster was identified in ETS-13 (Figure 6B) and was found to be similar to JSC-1 cluster except for a genomic inversion in the region comprised between *psbH2* and *psbA3* (Figure 6B). The gene cluster is greatly rearranged among the cyanobacteria groups (Gan et al., 2015), and it is interesting to notice the divergence with *Kovacikia minuta*. A species strictly related from a phylogenetic point of view. Furthermore, the LoLiP operon (Supplementary Table S7), identified in a thermofilic *Synechoccoccus* strain is present in the genome of four known FarLiP strains and in *Leptolyngbya* sp. PCC 6406 (Gan et al., 2015), however, was not found in ETS-13 genome. As reported by Soulier et al., 2020, *apcD4* and *apcB3* are similar to FarLiP allophycocyanin genes; this finding was confirmed in ETS-13 where targets with high sequence identity were identified as *apcD3* and *apcB2*. Moreover, genes related to CCA, in particular type II (CA2) and type III (CA3) acclimation responses, were investigated (Supplementary Table S8). In detail, the CA2 genes *ccaS*, *ccaR*, and *cpcL* (Hirose et al., 2008) were not found, due to the absence of phycoerythrin. As expected, genes encoding for photoreceptor RcaE and regulators RcaF and RcaC, as well as photoreceptor DpxA (Wiltbank and Kehoe, 2019) were not identified in the genome, as assumed for CA2. Finally, the genes responsible for scytonemin and mycosporine-like amino acids (MAAs) were not identified in ETS-13 genome, indicating the impossibility for this thermotolerant cyanobacterium to synthesize these UV-absorbing compounds (Supplementary Table S9).

Taxonomic Treatment and Description of *Kovacikia euganea* Zampieri & La Rocca, sp. nov. (Figures 2A–F).

Description: filaments bright green in color; largely tangled and coiled into clusters; rarely straight and solitary; forming mats attached to surfaces or floating. Filaments isopolar, unbranched, not attenuated at their ends; sheath covering trichomes thin and colorless. Cells elongated 1.5 ± 0.1 μm in width and 2.4 ± 1 μm in length; no heterocysts, akinetes, or hormogonia formation observed; thylakoids disposed in a simple parietal arrangement; 4–6 thylakoids parallel in the periphery of the cells. Extracellular vesicles present between the outer membranes and sheaths. Phycocyanin and allophycocyanin synthesis; absence of phycoerythrin; carotenoids present are *β*-carotene, echinenone, nostoxanthin, and caloxanthin; chlorophyll *f* produced under far-red light; FaRLiP gene cluster present in the genome.

Etymology: The specific epithet “euganea” refers to the name of the collection area, the Euganean Thermal District.

Habitat: Ponds and tanks used for the maturation process of the mud in spas of the Euganean Thermal District. Present in microbial mats exposed to thermal water (40–55°C).

Type locality: Euganean Thermal District (45°21’N, 11°46′E), Abano Terme, Padova, Italy.

Living culture: A living culture of strain ETS-13, from which the holotype was derived, is available in the laboratory of N. La Rocca (University of Padova).

Holotype here designated: Resin-embedded sample of ETS-13 deposited at the Herbarium Patavinum (PAD), the herbarium of the Botanical Garden, University of Padova, with the deposition number A000841.

## 4 Conclusion

In the present study, a new species of the Leptolyngbyaceae family was isolated from thermal springs and described as a new species. A polyphasic approach was followed for the correct taxonomic classification of strain ETS-13 and the results indicated that it belongs to the genus *Kovacickia.* This supports the hypothesis proposed by Tang et al. (2022) that members of the genus, characterized by a cosmopolitan distribution, are specific to thermal habitats. Moreover, physiological assessment showed its capability to perform FaRLiP. This ability is in line with the environmental conditions typical of the microbial mats where light is enriched in far-red. However, FaRLiP has been demonstrated so far just in a limited number of species isolated worldwide, in different environments of distant geographical areas and belonging to different genera. Interestingly all other *Kovacikia* species so far tested for FaRLiP from both thermal and freshwater environments resulted capable of this acclimation, suggesting this could be a common feature of the genus, with *K. euganea* as the first species isolated in Italy. *K. euganea* is the most abundant cyanobacterium species of the Euganean Thermal District at water temperature higher than 45°C (Gris et al., 2020). Isolation and characterization of the species *K. euganea* could also represent a starting point to assess whether it is involved in the therapeutic efficacy of mud treatments.

## Data Availability

The datasets presented in this study can be found in online repositories. The data can be found at: https://www.ncbi.nlm.nih.gov/, BioProject PRJNA946549.

## Footnotes

^1^ https://alignmentviewer.org/
