# Supplementary material for: Kovacikia euganea sp. nov. (Leptolyngbyaceae, Cyanobacteria), a new chlorophyll f producing cyanobacterium from the Euganean Thermal District (Italy)
Source: Front Microbiol. 2025 Mar 10;16:1545008. doi: 10.3389/fmicb.2025.1545008 (PMC11931122; doi:10.3389/fmicb.2025.1545008)
Supplement: Supplementary file 1 [file Data_Sheet_1.docx]

Supplementary Material


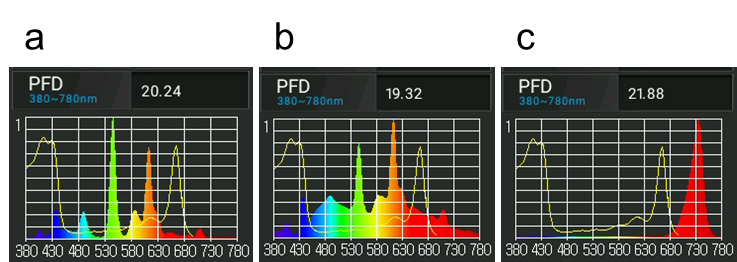


**Supplementary Figure 1.** Spectra of the lights used in the experiments: white light neon (a), solar light simulator (b), and far-red light (c) detected using LI-COR LI-180 spectrometer (Ecosearch Srl, Italy). The yellow line depicts the absorption spectrum of chlorophyll *a* with the peaks at 668 nm and 410-420 nm, from which it is possible to appreciate how the far-red light is shifted to longer wavelengths (from 700 to 750 nm) respect to the chlorophyll *a* peak.


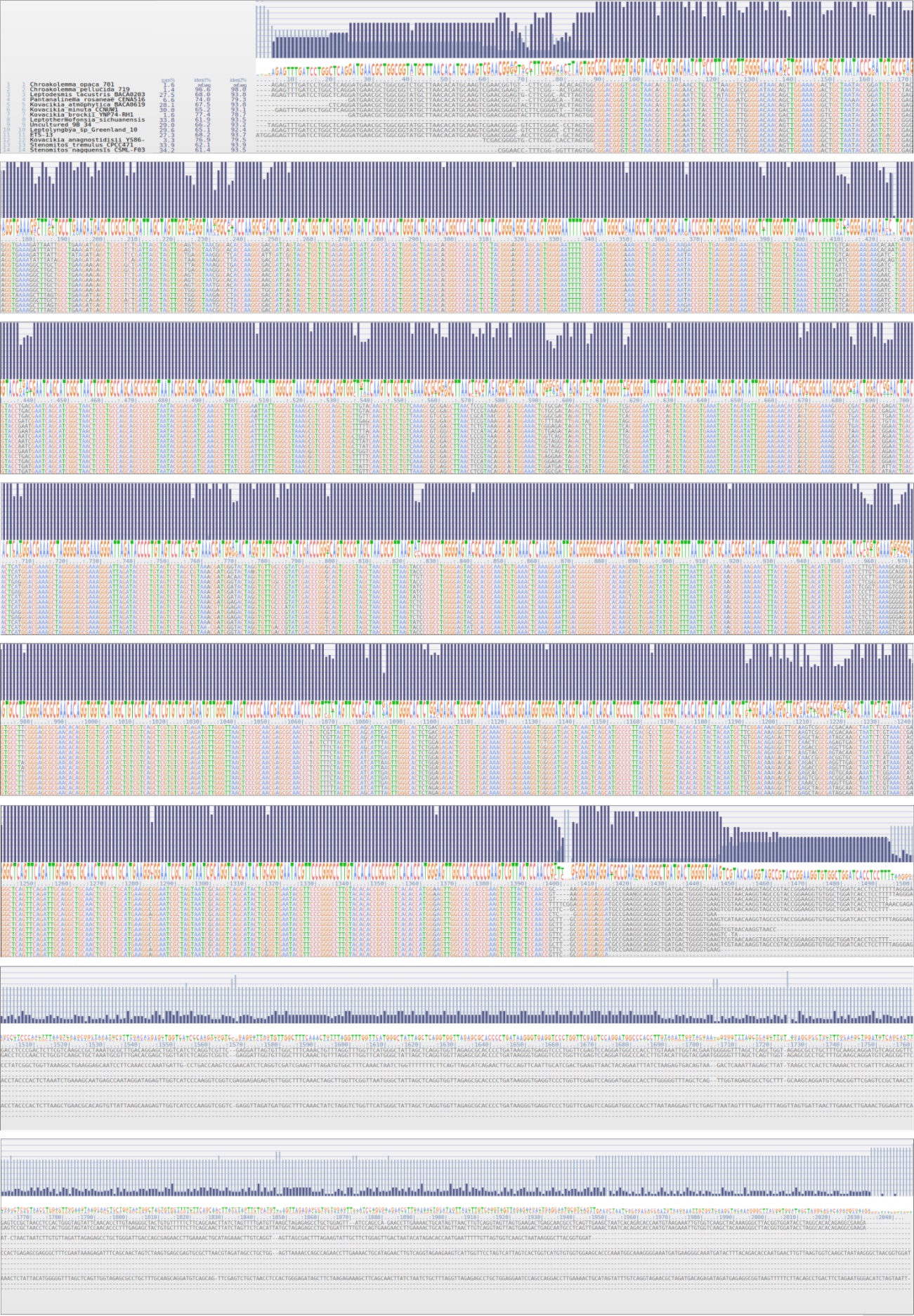


**Supplementary Figure S2** Multiple alignment of 16S rRNA sequences obtained using ETS-13 as query in BLAST N search, visualized using the software https://alignmentviewer.org/.

**
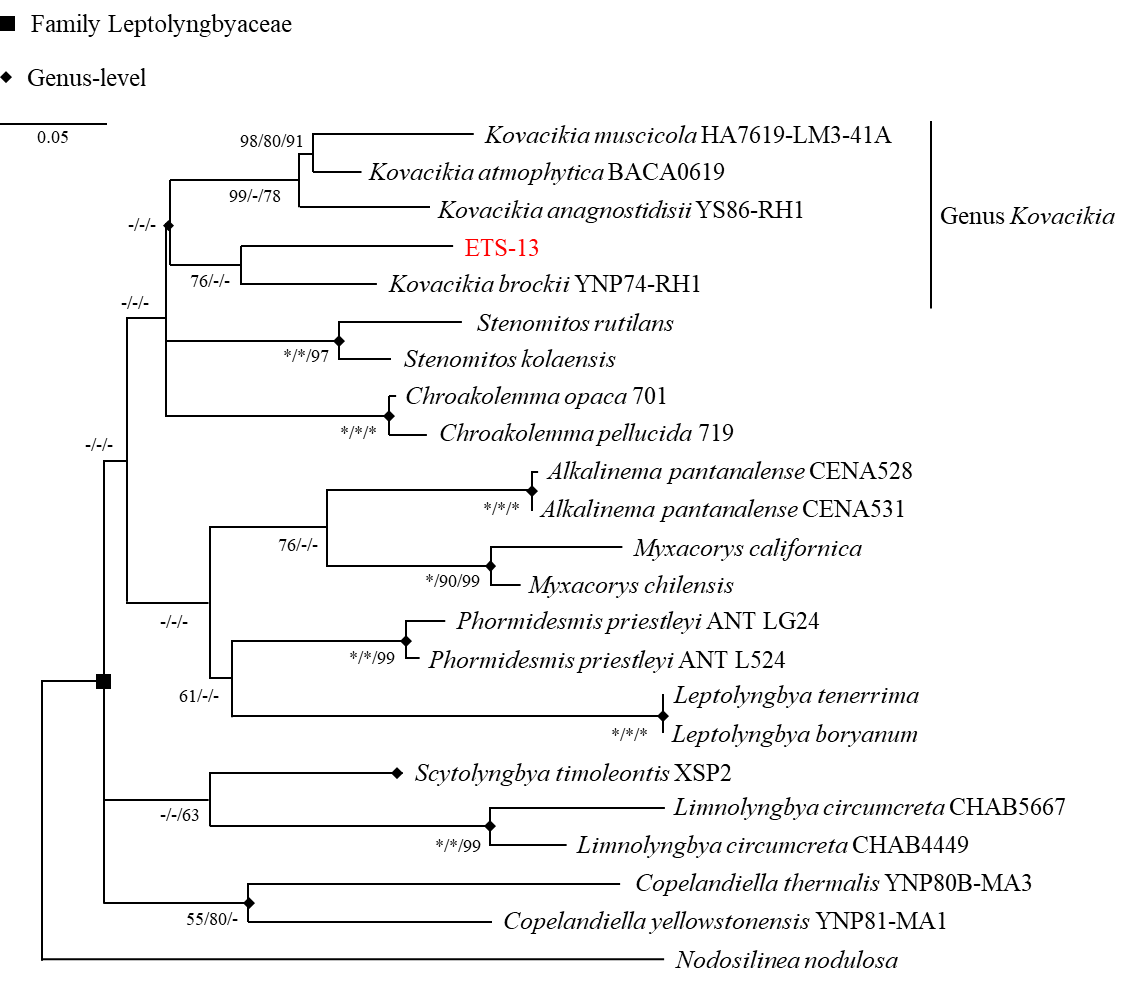
**

**Supplementary Figure 3** ML phylogenetic tree of 16S gene sequence inferred using GTR model. The tree with the highest log likelihood (-6877.83) is shown. The tree is drawn to scale, with branch lengths measured in the number of substitutions per site. The percentage of replicate trees in which the associated taxa clustered together in the bootstrap tests (1000 replicates each) are shown beside the branches in the order ML/NJ/MP. * = 100, - < 50


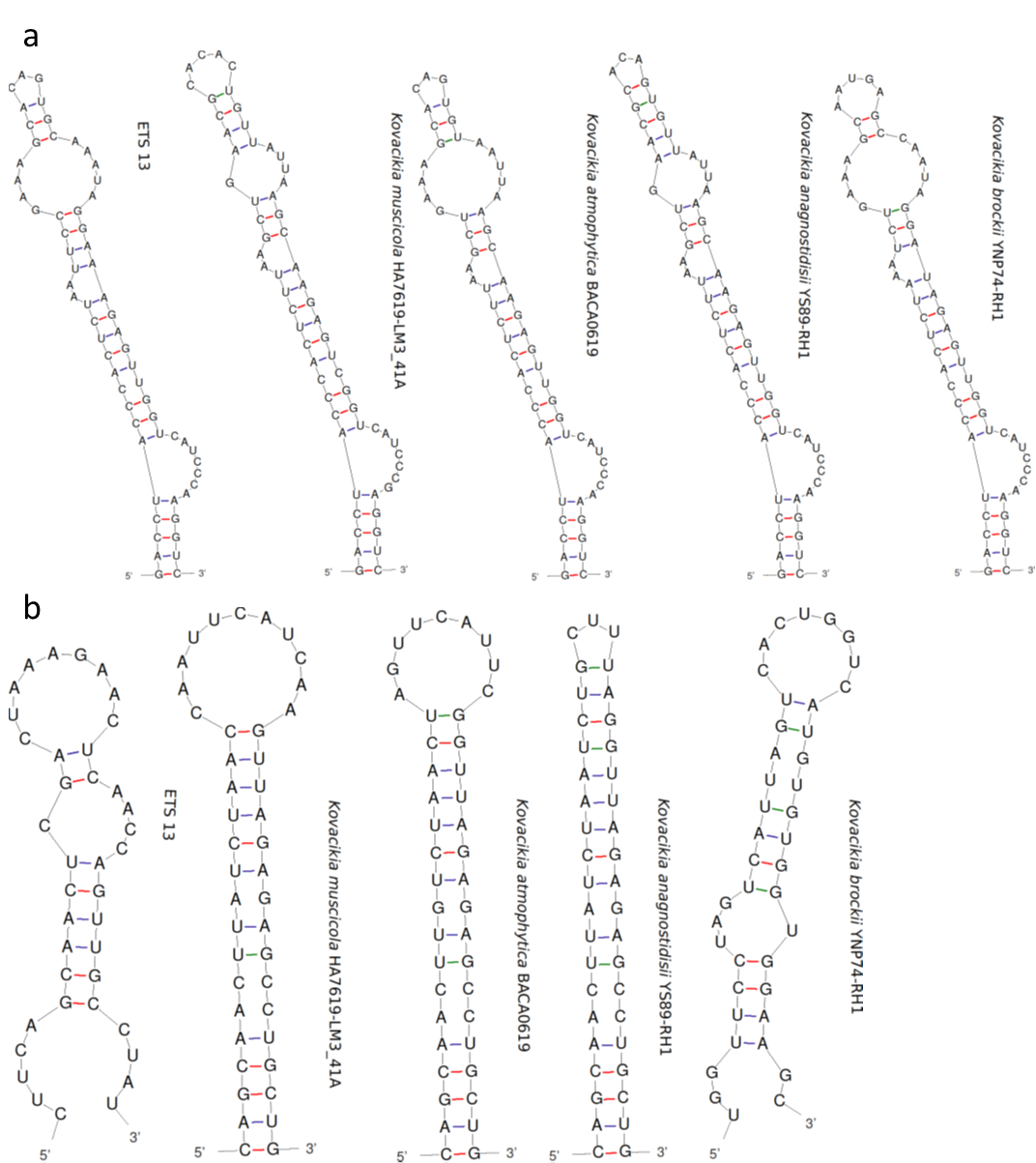


**Supplementary Figure 4** Comparison between the D1-D1’ region (a) and the boxB region (b) of strain ETS-13 and other members of the *Kovacikia* genus. Name of the species is reported near each structure.


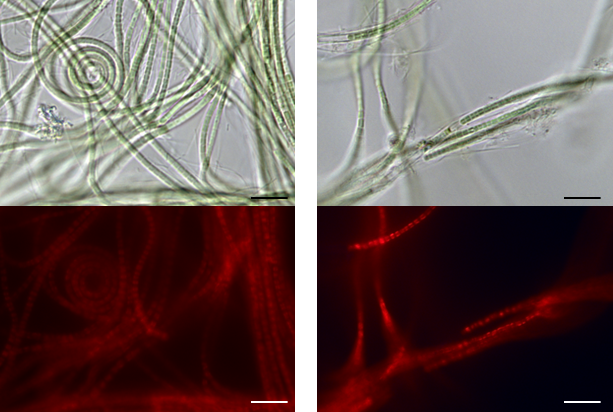


**Supplementary Figure 5** Optical microscopy images of ETS-13 cells grown in solar (left) or far-red light (right). Images were acquired after 21 days of growth in brightfield and using the autofluorescence of chlorophyll *a* to check cells viability. Bar scale: 10 µm.


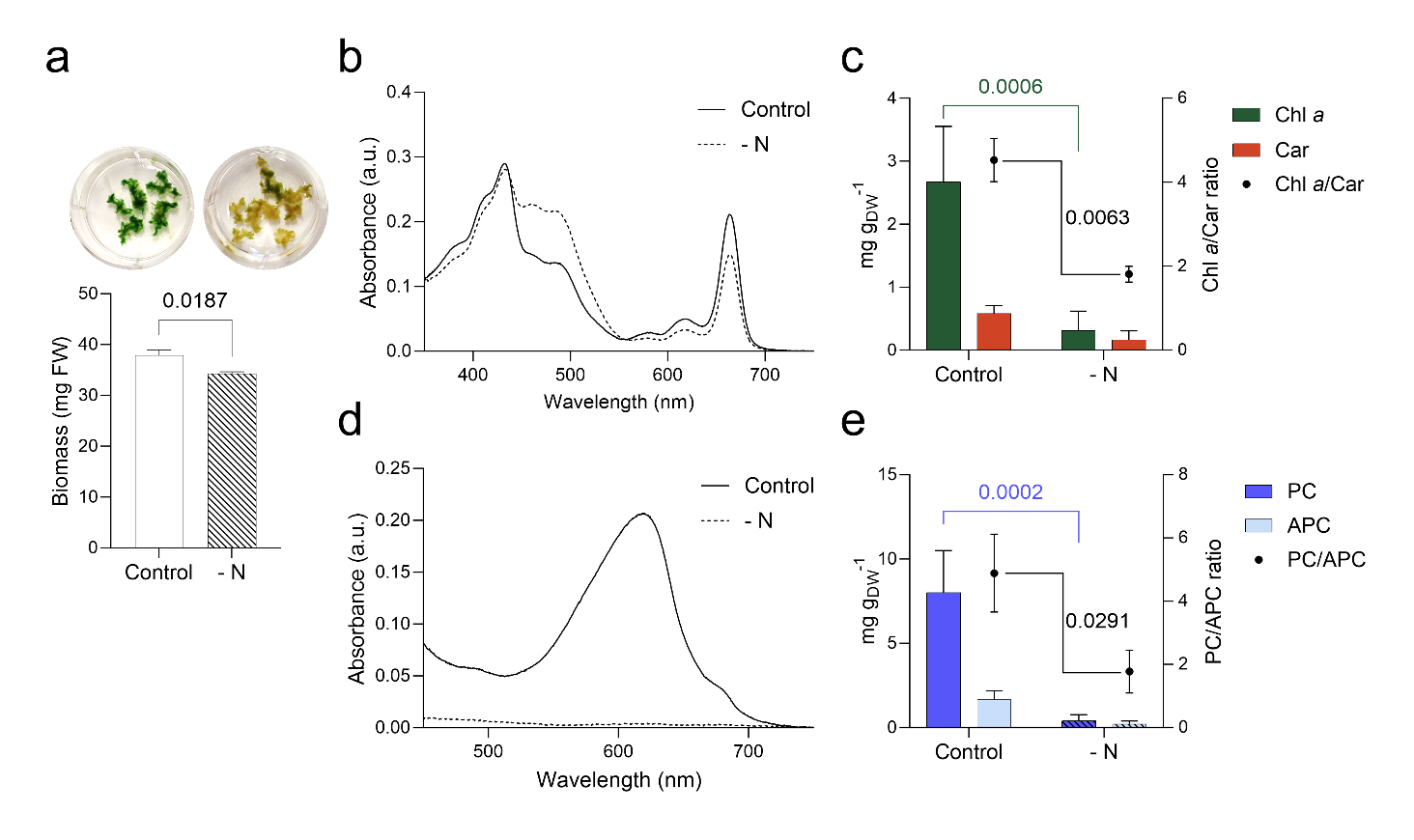


**Supplementary Figure 6** Growth of ETS-13 on a nitrogen-depleted medium (-N), compared to BG11 (Control). (a) Biomass reported as fresh weight (FW) at the end of the experiment, on top of the bars a representative picture of the cultures is present. (b) Lipid-soluble extract spectrum from 350 to 750 nm and (c) quantification of chlorophylls (Chl *a*) and carotenoids (Car). The ratio between the two components is also reported. (d) Water-soluble extract spectrum from 450 to 750 nm and (c) quantification of phycocyanin (PC) and allophycocyanin (APC). The ratio between the two phycobiliproteins is also reported. Average calculated from three biological replicates and the corresponding standard deviations are plotted. Statistics used: unpaired t test with Welch's correction for biomass and ratios, two-way ANOVA with Šídák's multiple comparisons test for pigment contents. p values are reported.

To test the ability of ETS-13 to fix atmospheric nitrogen, it was cultivated in standard medium BG11 as control condition, or using BG11_0_, which lacks the source of nitrogen present in BG11 as NaNO_3_. Tests were carried out using the optimal parameters previously determined for species growth: 30 °C and 30 µmol photons m^-2^ s^-1^. After 14 days of growth, the reduction in biomass in BG11_0_ was found to be statistically significant and the identification of cells turning from green to orange confirmed that cells cannot cope with this nutrient deficiency [1]. As confirmation, chlorophylls and carotenoids content drastically decreased as absolute abundance, while an increase in the relative abundance of carotenoids with respect to chlorophylls was identified during nitrogen starvation [2].

**Table S1** Target sequences and identity values obtained using ETS-13 16S rRNA gene sequence as query in BLAST N search (accessed: June 2024). The NCBI Reference Sequence code is reported in blue.

| Strain | 1 | 2 | 3 | 4 | 5 | 6 | 7 | 8 | 9 | 10 | 11 | 12 | 13 | 14 |
| --- | --- | --- | --- | --- | --- | --- | --- | --- | --- | --- | --- | --- | --- | --- |
| 1. **ETS-13** |  |  |  |  |  |  |  |  |  |  |  |  |  |  |
| 2. *Leptolyngbya* sp. Greenland 10 (DQ431005.1) | **96.24** |  |  | | | | | | | | | | | |
| 3. L*eptothermofonsia sichuanensis* strain PKUAC-SCTE412 (NR_176518.1) | **95.42** | 96.39 |  | | | | | | | | | | | |
| 4. *Kovacikia anagnostidisii* strain YS86-RH1 (NR_189703.1) | **95.35** | 95.18 | 95.94 |  | | | | | | | | | | |
| 5. Uncultured cyanobacterium clone 9B-34 (JX298770.1) | **95.24** | 96.39 | 99.78 | 95.63 |  | | | | | | | | | |
| 6. *Kovacikia atmophytica* strain BACA0619 (NR_191035.1) | **94.78** | 95.73 | 96.09 | 97.48 | 95.76 |  | | | | | | | | |
| 7. *Kovacikia brockii* strain YNP74-RH1 (NR_189704.1) | **94.74** | 95.51 | 97.34 | 94.91 | 96.93 | 95.17 |  | | | | | | | |
| 8. *Stenomitos tremulus* strain CPCC 471 (NR_176479.1) | **94.53** | 94.24 | 93.97 | 95.34 | 93.66 | 95.20 | 93.36 |  | | | | | | |
| 9. *Kovacikia minuta* strain CCNU0001 (NR_176592.1) | **94.27** | 95.69 | 94.99 | 96.15 | 94.76 | 96.67 | 95.24 | 94.94 |  | | | | | |
| 10. *Chroakolemma opaca* 701 (NR_177771.1) | **94.07** | 92.63 | 93.58 | 93.92 | 93.59 | 93.88 | 93.05 | 94.51 | 93.37 |  | | | | |
| 11. *Stenomitos nagquensis* strain CSML-F035 (NR_186900.1) | **94.12** | 93.45 | 93.42 | 95.33 | 92.18 | 95.15 | 93.36 | 99.24 | 94.71 | 94.20 |  | | | |
| 12. *Chroakolemma pellucida* 719 (NR_177773.1) | **93.94** | 92.70 | 93.66 | 94.08 | 93.52 | 93.88 | 92.98 | 94.51 | 93.51 | 99.46 | 94.37 |  | | |
| 13. *Leptodesmis lacustris* strain BACA0203 (NR_189177.1) | **93.86** | 96.32 | 93.05 | 93.77 | 93.02 | 93.67 | 93.11 | 93.79 | 94.33 | 93.80 | 94.52 | 94.07 | | |
| 14. *Pantanalinema rosaneae* (NR_177890.1) | **93.78** | 94.01 | 93.96 | 94.13 | 93.92 | 93.57 | 94.84 | 93.94 | 93.85 | 93.52 | 94.59 | 93.44 | 93.44 | |

**Table S2** Strains known or predicted (indicated with an *) to perform FaRLiP.

| **Order** | **Strain** | **Isolation habitat** | **Reference** |
| --- | --- | --- | --- |
| Chroococcales | *Chlorogloeopsis fritschii* PCC 9212 | Thermal spring (Spain) | [3] |
|  | *Chlorogloeopsis fritschii* PCC 6912 | Soil (India) | [4] |
|  | *Cyanosarcina cf burmensis* CCALA 770 * |  | [5] |
| Chroococcidiopsidales | *Chroococcidiopsis cubana* SAG 39.79 | Dry soil (Cuba) | [5] |
|  | *Chroococcidiopsis cubana* CCALA 040, 041, 042, 043, 045 * | Mineral spring (Cuba) | [6] |
|  | *Chroococcidiopsis thermalis* PCC 7203 | Soil (Germany) | [7] |
|  | *Chroococcidiopsis thermalis* CCALA 048 | (Cuba) | [6] |
|  | *Chroococcidiopsis thermalis* CCALA 050 | (Slovakia) | [6] |
|  | *Chroococcidiopsis* sp. CCALA 046 *, 927 | (Romania) | [6] |
|  | *Chroococcidiopsis* sp. CCMEE 010, 012 | Negev desert (Israel) | [8] |
|  | *Chroococcidiopsis* sp. CCMEE 130 | Cryptoendolithic on sandstone (USA) | [6] |
|  | *Chroococcidiopsis* sp. CCMEE 569, 570, 584 | Hypolithic, Gobi desert (Mongolia) | [6] |
|  | *Chroococcidiopsis* sp. CCNUC1, CCNUC2, CCNUC3, CCNUM1 | Epiphyte, arid limestone (China) | [6] |
|  | *Chroococcidiopsis* sp. SAG 2025 | Endolithic in coral rock (USA) | [6] |
| Nostocales | *Calothrix* PCC 7507 | Sphagnum bog (Switzerland) | [3] |
|  | *Calothrix* NIES-3974 * |  | [5] |
|  | *Calothrix parasitica* NIES-267 * |  | [5] |
|  | *Fischerella* JSC-11 * | Yellowstone hot spring (USA) | [3] |
|  | *Fischerella major* NIES-592 * |  | [5] |
|  | *Fischerella major* NIES-4106 * |  | [5] |
|  | *Fischerella major* NIES-3754 * |  | [5] |
|  | *Fischerella musicola* PCC 7414 * | Hot spring (New Zealand) | [3] |
|  | *Fischerella* PCC 9605 * | Freshwater (Israel) | [3] |
|  | *Fisherella thermalis* PCC 7521 | Yellowstone hot spring (USA) | [9] |
|  | *Mastigocoleus testarum* BC008 | Marine snail shell (Puerto Rico) | [10] |
|  | *Mastigocoleus laminosus* SAG 4.84 |  | [5] |
| Pleurocapsales | *Hydrococcus rivularis* NIES-593 * |  | [5] |
|  | *Pleurocapsa minor* PCC 7327 * | Thermal spring (USA) | [3] |
|  | *Pleurocapsa* sp. CCALA 161* |  | [5] |
| Synechococcales | *Aphanocapsa* sp. KC1 | Lake Biwa (Japan) | [11] |
|  | *Aphanocapsa* sp. KC1 (-like) | Outside cave (Australia) | [12] |
|  | ETS-13 | Euganean Thermal District (Italy) | This study |
|  | *Halomicronema hongdechloris* C2206 | Stromatolites (Australia) | [13] |
|  | *Kovacikia minuta* | Hydrophyte in a pond (China) | [14] |
|  | *Leptolyngbya* CCM4 | Soil mat (Mexico) | [15] |
|  | *Leptolyngbya* sp. JSC-1 | Yellowstone hot spring (USA) | [16] |
|  | *Leptothermofonsia sichuanensis* PKUAC-SCTE412 | Lotus lake hot springs (China) | [17] |
|  | *Oscillatoriales cyanobacterium* JSC-12 | Yellowstone hot spring (USA) | [3] |
|  | *Synechococcus* sp PCC 7335 | Snail shell, intertidal zone (Mexico) | [18] |
| Unclassified | Cyanobacterium TDX16 * |  | [5] |

**Table S3 N**umber of genes assigned to each COG category.

| **Cluster of Orthologous Groups** | **Number of genes** |
| --- | --- |
| Function unknown | 1034 |
| Replication and repair | 329 |
| Cell wall/membrane/envelope biogenesis | 312 |
| Energy production and conversion | 268 |
| Amino acids metabolism and transport | 243 |
| Signal transduction | 232 |
| Transcription | 224 |
| Inorganic ion transport and metabolism | 212 |
| Post-translational modification | 189 |
| Translation | 180 |
| Carbohydrate metabolism and transport | 179 |
| Coenzyme metabolism | 158 |
| Lipid metabolism | 89 |
| Nucleotide metabolism and transport | 88 |
| Intracellular trafficking and secretion | 86 |
| Secondary structure | 71 |
| Cell cycle control and mitosis | 64 |
| Defense mechanism | 56 |
| Cell motility | 41 |
| RNA processing and modification | 12 |
| Chromatin structure and dynamics | 4 |

**Table S4** Genes involved in the synthesis of four cyanotoxins investigated in this study.

| **Description** | **Gene** | **GenBank accession:** | **Organism** |
| --- | --- | --- | --- |
| Mycrocystin | *mycE* | CCI23892 | *Microcystis aeruginosa* PCC 9808 |
|  |  | FJ393328 | *Microcystis* sp. CYN10 |
| Cylindrospermopsin | *cyrC* | LC423023 | *Cylindrospermopsis raciborskii* NUSPBC7 |
|  |  | AHN91608 | *Raphidiopsis curvata* HB1 |
|  | *cyrB* | MN909338 | Uncultured cyanobacterium |
|  |  | ADY11245 | Uncultured cyanobacterium |
|  | *cyrA* | JN014841 | *Cylindrospermopsis raciborskii* CCMP1973 |
|  |  | AEM00322 | *Cylindrospermopsis raciborskii* CCMP1973 |
|  | *cyrJ* | KY550407 | *Cylindrospermopsis raciborskii* AWQC-CYP011K |
|  |  | ASU10954 | *Cylindrospermopsis raciborskii* AWQC-CYP011K |
| Saxitoxin | *sxtA* | LC549066 | *Alexandrium tamarense* |
|  |  | SAQ71186 | *Aphanizomenon gracile* NIVA-CYA 676 |
|  | *sxtB* | KC894588 | *Cylindrospermopsis raciborskii* CENA305 |
|  |  | SAQ71185 | *Aphanizomenon gracile* NIVA-CYA 676 |
|  | *sxtI* | KJ123835 | *Aphanizomenon gracile* UAM531 |
|  |  | SAQ71203 | *Aphanizomenon gracile* NIVA-CYA 676 |
| Lyngbyatoxin-A | *ltxA* | AAT12283 | *Lyngbya majuscula* |
|  | *ltxB* | AAT12284 | *Lyngbya majuscula* |
|  | *ltxC* | AAT12285 | *Lyngbya majuscula* |
|  | *ltxD* | AAT12286 | *Lyngbya majuscula* |

**Table S5** Protein encoding genes performing atmospheric N_2_ fixation.

| **Description** | **Gene** | **GenBank accession** | **Organism** |
| --- | --- | --- | --- |
| Nitrogenase molybdenum-iron cofactor biosynthesis protein | *nifE* | BAU44872 | *Leptolyngbya* sp. O-77 |
|  |  | AAA87947 | *Nostoc* sp. PCC 7120 |
|  |  | AFW96196 | *Anabaena* sp. 90 |
|  | *nifN* | WP_068515280 | *Leptolyngbya* sp. O-77 |
|  |  | AAA87948 | *Nostoc* sp. PCC 7120 |
|  |  | QFZ11110 | *Anabaena* sp. YBS01 |
| Nitrogenase iron protein | *nifH* | WP_068515290 | *Leptolyngbya* sp. O-77 |
|  |  | CAA83510 | *Nostoc* sp. PCC 6720 |
|  |  | AFW96085 | *Anabaena* sp. 90 |
|  | *nifD* | WP_068515287 | *Leptolyngbya* sp. O-77 |
|  |  | CAA24730 | *Nostoc* sp. PCC 7120 |
|  |  | AFW96173 | *Anabaena* sp. 90 |
|  | *nifK* | WP_068515285 | *Leptolyngbya* sp. O-77 |
|  |  | ANQ45517 | *Nostoc flagelliforme* NX-09 |
|  |  | AFW96195 | *Anabaena* sp. 90 |
| Nitrogenase cofactor biosynthesis protein | *nifB* | WP_015137586. | *Nostoc* sp. PCC 7524 |
|  | *nifS* | AAA22006 | *Nostoc* sp. PCC 7120 |
|  | *nifU* | AAA22007 | *Nostoc* sp. PCC 7120 |
|  | *nifX* | AAA87949 | *Nostoc* sp. PCC 7120 |
| Nitrogen fixation related protein | *hesA* | BAB73389 | *Nostoc* sp. PCC 7120 |
| Heterocyst ferredoxin | *fdxH* | BAB73387 | *Nostoc* sp. PCC 7120 |

**Table S6** Annotation of the genes forming the cluster involved in far-red light photoacclimation; the proteins reported refer to *Chlorogloeopsis fritschii* PCC6912. In the bottom part of the table are reported the protein sequences of ETS-13.

| **Description** | **Protein** | **GenBank accession** | **ETS-13 name** |
| --- | --- | --- | --- |
| Photosystem I reaction center subunit VIII | psaI2 | WP_016873408 | psaI2_ETS-13 |
| Photosystem I reaction center subunit XI | psaL2 | WP_016873409 | psaL2_ETS-13 |
| Photosystem I core protein | psaB2 | WP_016873410 | psaB2_ETS-13 |
| Photosystem I P700 chlorophyll *a* apoprotein A1 | psaA2 | WP_016873411 | psaA2_ETS-13 |
| Phytochrome photoreceptor | rfpA | WP_016873415 | rfpA_ETS-13 |
| Phytochrome response regulator | rfpB | WP_016879258 | rfpB_ETS-13 |
| Phytochrome response regulator | rfpC | WP_016873417 | rfpC_ETS-13 |
| Chlorophyll *f* synthase | psbA4 | WP_016873418 | psbA4_ETS-13 |
| Photosystem II q(b) protein | psbA3 | WP_016873419 | psbA3_ETS-13 |
| Allophycocyanin subunit alpha-B | apcD3 | WP_016873420 | apcD3_ETS-13 |
| Allophycocyanin subunit beta | apcB2 | WP_016873421 | apcB2_ETS-13 |
| Allophycocyanin subunit alpha-B | apcD2 | WP_016873422 | apcD2_ETS-13 |
| Phycobilisome rod-core linker polypeptide | apcE2 | WP_016873423 | apcE2_ETS-13 |
| Allophycocyanin subunit alpha-B | apcA2 | WP_016873424 | apcA2_ETS-13 |
| Photosystem II D2 protein (photosystem q(a) protein) | psbD3 | WP_016873425 | psbD3_ETS-13 |
| Photosystem II reaction center protein CP43 | psbC2 | WP_016873426 | psbC2_ETS-13 |
| Photosystem II chlorophyll-binding protein CP47 | psbB2 | WP_016873427 | psbB2_ETS-13 |
| Photosystem II protein" | psbH2 | WP_016873428 | psbH2_ETS-13 |
| Photosystem I reaction center subunit III | psaF2 | WP_016879260 | psaF2_ETS-13 |
| Photosystem I reaction center subunit IX | psaJ2 | WP_016873431 | psaJ2_ETS-13 |

**Table S7** Genes in the polycistronic unit involved in low-light photoacclimation (LoLiP).

| **Description** | **Gene** | **GenBank accession** | **Organism** |
| --- | --- | --- | --- |
| Allophycocyanin subunit alpha-B | *apcD4* | WP_016874155 | *Chlorogloeopsis fritschii* |
| Hypothetical protein | *apcB3* | WP_016874154 | *Chlorogloeopsis fritschii* |
| Chlorophyll a/b binding light-harvesting protein | *isiX* | NZ_RSCJ01000001 | *Chlorogloeopsis fritschii* |

**Table S8** Genes coding for phytochrome photoreceptors involved in chromatic acclimation (CA2 and CA3) responses.

| **Description** | **Gene** | **GenBank accession** | **Organism** |
| --- | --- | --- | --- |
| Cyanobacteriochrome activated by red and green light (CA2) | *ccaS* | BAK51774 | *Synechocystis* sp. PCC 6803 |
|  |  | BDA72134 | *Calothrix* sp. PCC 7716 |
| Regulator gene (CA2) | *ccaR* | ABM67667 | *Synechocystis* sp. PCC 6803 |
|  |  | BDA72138 | *Calothrix* sp. PCC 7716 |
| Phycobiliprotein (CA2) | *cpcL* | P74625 | *Synechocystis* sp. PCC 6803 |
| Cyanobacteriochrome dominant in red light (CA3) | *rcaE* | U59741 | *Fremyella diplosiphon* |
| Response regulator (CA3) | *rcaF* | AF002707 | *Fremyella diplosiphon* Fd33 |
| Response regulator (CA3) | *rcaC* | KEI67872 | *Planktothrix agardhii* NIVA-CYA 126/8 |
| Cyanobacteriochrome dominant in yellow to green light (CA3) | *dpxA* | WP_045867496 | unclassified *Tolypothrix* |

**Table S9** Genes and protein sequences for scytonemin and mycosporine-like amino acids (MAAs).

| **Description** | **Gene** | **GenBank accession** | **Organism** |
| --- | --- | --- | --- |
| Scytonemin biosynthesis cyclase/decarboxylase | *scyC* | WP_196524196 | *Nostoc commune* |
|  |  | WP_198124104 | *Amazonocrinis nigriterrae* |
| Putative scytonemin biosynthesis protein | *scyC* | FJ605304 | *Chlorogloeopsis* sp. Cgs-089 |
| Mycosporine lysine biosynthetic gene cluster | *myl* | KU376485 | *Cylindrospermum stagnale* PCC 7417 |
| 3-dehydroquinate synthetase | *mylA* | AFZ23628 |  |
| Putative O-methyltransferase | *mylB* | AFZ23629 |  |
| Putative ATP-grasp enzyme | *mylC* | AFZ23631 |  |
| hypothetical protein Cylst_1343 | *mylD* | AFZ23632 |  |
| ATP-grasp enzyme, D-alanine-D-alanine ligase | *mylE* | AFZ23630 |  |

References

[1] M.M. Allen, A.J. Smith, Nitrogen chlorosis in blue-green algae, Arch Mikrobiol 69 (1969) 114–120. https://doi.org/10.1007/BF00409755.

[2] I.C. de Loura, J.P. Dubacq, J.C. Thomas, The Effects of Nitrogen Deficiency on Pigments and Lipids of Cyanobacteria, Plant Physiol 83 (1987) 838–843. ttps://doi.org/10.1104/pp.83.4.838.

[3] F. Gan, G. Shen, D.A. Bryant, Occurrence of far-red light photoacclimation (FaRLiP) in diverse cyanobacteria, Life 5 (2015) 4–24. https://doi.org/10.3390/life5010004.

[4] R.L. Airs, B. Temperton, C. Sambles, G. Farnham, S.C. Skill, C.A. Llewellyn, Chlorophyll *f* and chlorophyll *d* are produced in the cyanobacterium *Chlorogloeopsis fritschii* when cultured under natural light and near-infrared radiation, FEBS Lett 588 (2014) 3770–3777. https://doi.org/10.1016/j.febslet.2014.08.026.

[5] L.A. Antonaru, T. Cardona, A.W.D. Larkum, D.J. Nürnberg, Global distribution of a chlorophyll *f* cyanobacterial marker, ISME Journal 14 (2020) 2275–2287. https://doi.org/10.1038/s41396-020-0670-y.

[6] L.A. Antonaru, V.M. Salinger, P. Jung, G. Di Stefano, N.D. Sanderson, L. Barker, D.J. Wilson, B. Büdel, D.P. Canniffe, D. Billi, D.J. Nürnberg, Common loss of far-red light photoacclimation in cyanobacteria from hot and cold deserts: a case study in the Chroococcidiopsidales, ISME Journal (2023). https://doi.org/10.1038/s43705-023-00319-4.

[7] D.J. Nürnberg, J. Morton, S. Santabarbara, A. Telfer, P. Joliot, L.A. Antonaru, A. v. Ruban, T. Cardona, E. Krausz, A. Boussac, A. Fantuzzi, A. William Rutherford, Photochemistry beyond the red limit in chlorophyll *f*–containing photosystems, Science (1979) 360 (2018) 1210–1213. https://doi.org/10.1126/science.aar8313.

[8] D. Billi, A. Napoli, C. Mosca, C. Fagliarone, R. De Carolis, A. Balbi, M. Scanu, V.M. Selinger, L.A. Antonaru, D.J. Nürnberg, Identification of far-red light acclimation in an endolithic *Chroococcidiopsis* strain and associated genomic features: Implications for oxygenic photosynthesis on exoplanets, Front Microbiol (2022). https://doi.org/10.3389/fmicb.2022.933404.

[9] G. Hastings, H. Makita, N. Agarwala, L. Rohani, G. Shen, D.A. Bryant, Fourier transform visible and infrared difference spectroscopy for the study of P700 in photosystem I from *Fischerella thermalis* PCC 7521 cells grown under white light and far-red light: Evidence that the A –1 cofactor is chlorophyll *f*, Biochim Biophys Acta Bioenerg 1860 (2019) 452–460. https://doi.org/10.1016/j.bbabio.2019.04.002.

[10] E.L. Ramírez-Reinat, F. Garcia-Pichel, Characterization of a marine cyanobacterium that bores into carbonates and the redescription of the genus *Mastigocoleus*, J Phycol 48 (2012) 740–749. https://doi.org/10.1111/j.1529-8817.2012.01157.x.

[11] H. Miyashita, Discovery of Chlorophyll *d* in *Acaryochloris marina* and Chlorophyll *f* in a Unicellular Cyanobacterium, Strain KC1, Isolated from Lake Biwa, J Phys Chem Biophys 4 (2014) 1–9. https://doi.org/10.4172/2161-0398.1000149.

[12] L. Behrendt, A. Brejnrod, M. Schliep, S.J. Sørensen, A.W.D. Larkum, M. Kühl, Chlorophyll *f*-driven photosynthesis in a cavernous cyanobacterium, ISME Journal 9 (2015) 2108–2111. https://doi.org/10.1038/ismej.2015.14.

[13] M. Chen, Y. Li, D. Birch, R.D. Willows, A cyanobacterium that contains chlorophyll *f* - A red-absorbing photopigment, FEBS Lett 586 (2012) 3249–3254. https://doi.org/10.1016/j.febslet.2012.06.045.

[14] L.Q. Shen, Z.C. Zhang, J.L. Shang, Z.K. Li, M. Chen, R. Li, B.S. Qiu, *Kovacikia minuta* sp. nov. (Leptolyngbyaceae, Cyanobacteria), a new freshwater chlorophyll *f*-producing cyanobacterium, J Phycol 58 (2022) 424–435. https://doi.org/10.1111/jpy.13248.

[15] C. Gómez-Lojero, L.E. Leyva-Castillo, P. Herrera-Salgado, J. Barrera-Rojas, E. Ríos-Castro, E.B. Gutiérrez-Cirlos, Leptolyngbya CCM 4, a cyanobacterium with far-red photoacclimation from Cuatro Ciénegas Basin, México, Photosynthetica 56 (2018) 342–353. https://doi.org/10.1007/s11099-018-0774-z.

[16] I.I. Brown, D.A. Bryant, D. Casamatta, K.L. Thomas-Keprta, S.A. Sarkisova, G. Shen, J.E. Graham, E.S. Boyd, J.W. Peters, D.H. Garrison, D.S. McKay, Polyphasic characterization of a thermotolerant siderophilic filamentous cyanobacterium that produces intracellular iron deposits, Appl Environ Microbiol 76 (2010) 6664–6672. https://doi.org/10.1128/AEM.00662-10.

[17] J. Tang, M.R. Shah, D. Yao, Y. Jiang, L. Du, K. Zhao, L. Li, M. Li, M.M. Waleron, M. Waleron, K. Waleron, M. Daroch, Polyphasic Identification and Genomic Insights of *Leptothermofonsia sichuanensis* gen. sp. nov., a Novel Thermophilic Cyanobacteria Within Leptolyngbyaceae, Front Microbiol 13 (2022). https://doi.org/10.3389/fmicb.2022.765105.

[18] M.Y. Ho, F. Gan, G. Shen, D.A. Bryant, Far-red light photoacclimation (FaRLiP) in *Synechococcus* sp. PCC 7335. II.Characterization of phycobiliproteins produced during acclimation to far-red light, Photosynth Res 131 (2017) 187–202. https://doi.org/10.1007/s11120-016-0303-5.
